# Alternative approaches for monitoring and evaluation of lymphatic filariasis following mass drug treatment with ivermectin, diethylcarbamazine and albendazole in East New Britain Province, Papua New Guinea

**DOI:** 10.1371/journal.pntd.0012128

**Published:** 2025-01-27

**Authors:** Krufinta Bun, Benedict Mode, Melinda Susapu, Joyceline Salo, Catherine Bjerum, Michael Payne, Daniel Tisch, Makoto Sekihara, Emanuele Giorgi, Gary J. Weil, Peter U. Fischer, Leanne Robinson, Moses Laman, Christopher L. King

**Affiliations:** 1 Department of Pathology, Center for Global Health and Disease, Case Western Reserve University, Cleveland, Ohio, United States of America; 2 Provincial Health Department, Kokopo, East New Britain Province, Papua New Guinea; 3 Papua New Guinea Department of Health, Port Moresby, Papua New Guinea; 4 Papua New Guinea Institute of Medical Research, Goroka, Papua New Guinea; 5 Japan International Cooperation Agency (JICA), Tokyo, Japan; 6 Centre for Health Informatics, Computing and Statistics, Lancaster University, Lancaster, United Kingdom; 7 Infectious Diseases Division, Department of Medicine, Washington University, St. Louis, Missouri, United States of America; 8 Burnet Institute, Melbourne, Victoria, Australia; 9 Veterans Affairs Medical Center, Cleveland, Ohio, United States of America; National Institutes of Allergy and Infectious Diseases, NIH, UNITED STATES OF AMERICA

## Abstract

**Background:**

WHO recommends two annual rounds of mass drug administration (MDA) with ivermectin, diethylcarbamazine, and albendazole (IDA) for lymphatic filariasis (LF) elimination in treatment naïve areas that are not co-endemic for onchocerciasis such as Papua New Guinea (PNG). Whether two rounds of MDA are necessary or sufficient and the optimal sampling strategies and endpoints for stopping MDA remain undefined.

**Methods and findings:**

Two cross-sectional studies were conducted at baseline (N = 49 clusters or villages) and 12 months after mass drug administration (MDA) with IDA (N = 47 villages) to assess lymphatic filariasis (LF) by circulating filarial antigenemia (CFA) and microfilariae (Mf). Before MDA, children aged 6–9 years (N~50) and those  ≥  10 years (N~50) in each village were randomly sampled. Before MDA, the population mean prevalence of LF in East New Britain Province (ENBP), Papua New Guinea, was estimated using population proportionate sampling (PPS, N = 30) to be 59/2,561 (2.3%) CFA positive and 14/2,561 (0.6%) Mf positive. No children were Mf positive. However, LF infection was highly heterogeneous; 8 villages (26.7%) had a CFA prevalence >2%, and 7 villages (23.3%) had an Mf prevalence >1%. To identify sentinel villages with LF in areas under-sampled by PPS, 19 additional villages suspected to have LF were sampled, with 15 (79%) having >2% CFA prevalence and 7 (38%) >1% Mf (range 1**–**22%). Twenty-four villages were evaluated before and after MDA in age-matched adults ( ≥  18 years). Treatment reduced CFA prevalence by 34% and Mf prevalence by 90%. Post-MDA model-based geostatistics efficiently selected an additional 23 villages, of which 20 (87%) had a CFA prevalence  >  2%. None of these villages had >1% Mf. Post-MDA, two of four districts had no villages with >1% Mf.

**Conclusions:**

Model-based geostatistics was more effective than PPS in sampling high-risk LF sites in a heterogeneous area. Low LF prevalence and partial reduction of CFA limit children’s effectiveness as sentinels. A single round of high-coverage MDA with IDA achieved elimination targets in low-prevalence villages in PNG. Higher-prevalence areas will need additional MDA rounds, which could be targeted to smaller evaluation units to cut costs.

**Trial registration:**

Clinicaltrials.gov NCT04124250

## Author summary

**Why was this study done?:**

- WHO has targeted lymphatic filariasis (LF) for global elimination as a public health problem using mass drug administration (MDA) as the primary intervention strategy.
- The WHO recently modified recommendations for MDA for LF with a combination of three co-administered drugs: ivermectin, diethylcarbamazine, and albendazole. This study examined the impact of one round of MDA on LF infection parameters in Papua New Guinea that had not previously received MDA for LF and examined new methodologies for monitoring and surveillance.

**What did the researcher do and find?:**

- Before MDA, we randomly sampled sentinel villages using population proportional sampling of equal numbers of children 6–9 years and older children and adults using well-established LF infection parameters. Post-MDA, we selected sentinel villages using a geostatistical modeling design and focused on sampling adults.
- Population-proportional sampling estimated the overall LF mean prevalence. However, it led to the inappropriate conclusion that LF might be less important because PPS missed villages with high LF prevalence in less densely populated rural areas. Sampling children 6–9 years of age was inefficient because of low infection rates in this age group. One round of MDA with high coverage effectively reduced microfilaremia prevalence to very low levels in most sampled villages, but CFA prevalence decreased less dramatically.

**What do these findings mean?:**

- Geostatistical modeling and sampling adults for microfilaria are preferred methods for monitoring the impact of MDA with IDA in areas heterogeneous for LF rather than PPS. Sampling children to monitor LF elimination may not be a reliable indicator for stopping MDA, because CFA declines slowly after IDA, and children have low LF infection prevalence.
- Results from this study suggest that one round of high-coverage MDA may be sufficient to eliminate LF in areas with low baseline prevalence. Additional rounds of MDA can then be targeted to high-risk LF locations selected by geostatistical modeling, thus reducing program costs. The accuracy of geostatistical modeling in identifying LF-infected villages improves with high-quality baseline surveillance. This approach may be especially useful in areas like Papua New Guinea, where MDA is logistically challenging and costly.

## Introduction

Lymphatic filariasis (LF) is a disease caused by parasitic nematodes transmitted by mosquitoes. The infection leads to a spectrum of clinical outcomes that range from asymptomatic microfilaremia (parasite larvae circulating in the blood) to hydrocele, lymphedema, and elephantiasis [1]. Lymphatic filariasis is caused by the nematodes *Wuchereria bancrofti*, *Brugia malay*i, and *Brugia timori*, of which *W. bancrofti* accounts for over 90% of infections worldwide [2]. The World Health Organization (WHO) currently estimates that 883 million people in 44 countries are at risk for LF and that 15 million are disfigured and incapacitated by the disease [3]. The WHO started the Global Program to Eliminate Lymphatic Filariasis (GPELF) with the aim to eliminate LF by 2020 [2], which was later revised to 2030 [4]. The elimination strategy comprises community-wide mass drug administration (MDA) in endemic areas based on administrative units that often include a mix of areas with high and low LF endemicity. Mass drug treatment comprises annual single-dose therapy of albendazole (ALB) combined with diethylcarbamazine (DEC) in countries not co-endemic for onchocerciasis and ALB combined with ivermectin (IVM) in onchocerciasis co-endemic countries in sub-Saharan Africa that are not co-endemic for *Loa loa* [5]. Areas in Africa that are co-endemic for *Loa loa* and have high parasite loads should receive ALB only. The LF elimination strategy also includes morbidity management of advanced clinical cases. To eliminate LF, the current GPELF strategy recommends MDA with a minimum effective coverage of ≥65% of the total population for up to five annual rounds of MDA [6,7]. This strategy has been highly successful in many LF-endemic countries, resulting in 740 million people no longer requiring MDA. This amounts to a 52% reduction in the at-risk population worldwide [8]. However, this approach has been less successful in other LF endemic areas because of poor uptake of drugs in people with the highest risk of infection (e.g., adult men, migrants, and pregnant women), logistical challenges for drug delivery, and limited efficacy of the two-drug MDA regimens.

In 2017, the WHO endorsed the use of a single co-administered medication consisting of Ivermectin (IVM), Diethylcarbamazine (DEC), and Albendazole (ALB) (referred to as IDA) for the elimination of lymphatic filariasis (LF) in many areas outside of Africa [9]. This combination was more effective than using DEC plus ALB alone [10–15]. IDA is recommended in LF endemic areas that meet certain criteria: i) where onchocerciasis is not co-endemic, ii) where have not yet started MDA, iii) that have received fewer than four effective rounds of MDA, or iv) in areas where MDA results have been suboptimal [16]. Currently, WHO recommends two annual rounds of MDA with IDA in eligible countries with at least 65% epidemiological coverage.

One challenge associated with MDA is knowing when infection prevalence has been reduced to levels that no longer can support transmission so that MDA can be stopped. The current MDA stopping protocol uses transmission assessment surveys (TAS) to detect CFA in children 6–7 years of age as an indicator of recent transmission [17]. However, TAS can be insensitive to ongoing LF transmission in areas where infection prevalence is much lower in children than adults. Before proceeding to TAS, programs perform pre-TAS surveys to assess whether CFA prevalence is less than 2% or Mf prevalence is <1% in one sentinel and one spot-check site within an evaluation unit (EU). However, the focal distribution of LF represents an important limitation to this strategy; pre-TAS surveys may miss persistent infection when only some sites are sampled in large evaluation units. The detection of persistent CFA after two annual rounds of MDA with IDA is an unreliable marker for monitoring LF transmission because treatment does not kill all adult worms. It requires years to decline, long after MF prevalence has been reduced to levels that cannot sustain transmission [11]. However, CFA may be an informative variable for developing new tools for improving LF monitoring in EUs.

The Task Force for Global Health developed a new monitoring and evaluation approach to assess MDA with IDA to address these challenges. This approach was applied to East New Britain Province (ENBP), which received province-wide MDA for LF. The goals here are to i) evaluate the impact of one round of IDA on LF infection parameters, ii) provide evidence to support the adoption of new elimination criteria and endpoints using MDA with IDA, and iii) improve sampling strategies to understand better the focal nature of LF in the population and demonstrate the elimination of LF.

## Methods

### Ethics statement

The protocol was approved by the Medical Research Advisory Council of Papua New Guinea (MRAC 19.14) and Case Western Reserve University Institutional Review Board (STUDY20191141). Individual written informed consent was provided by adults aged 18 years and older. If the individuals could not read or write, a village member who could read or write witnessed and cosigned the informed consent form. Children’s participation required the consent of their parents and documented assent for children older than 12 years of age. Written consent was obtained from the parents or guardians of the children.

### Study site

**Fig 1:**
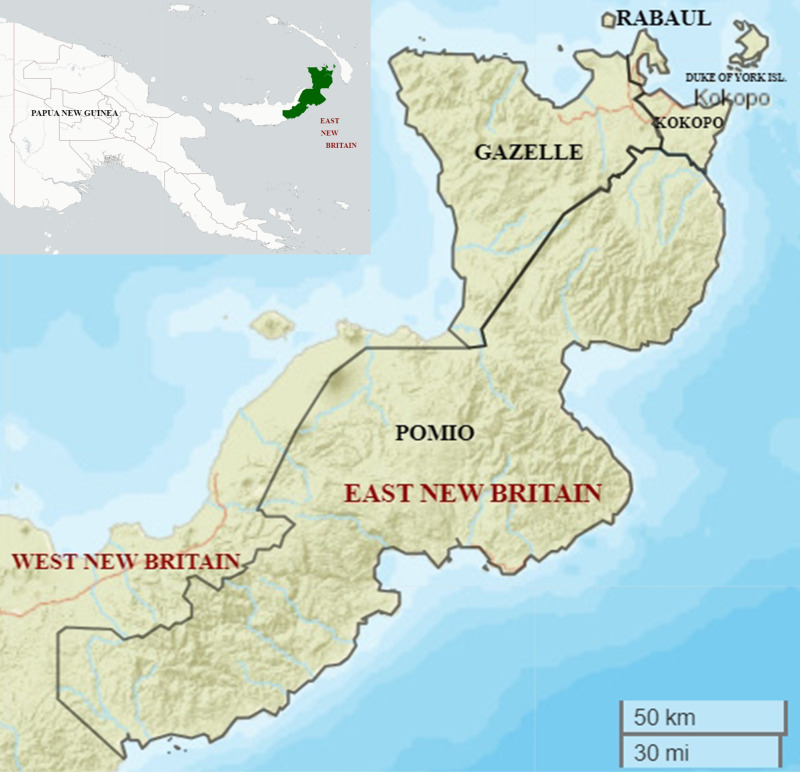
Map of East New Britain Province. The maps were made using R package, leaflet. Map data copyright from OpenStreetMap contributors and data is available under the Open Database License at https://www.openstreetmap.org/copyright. Basemap of PNG map (insert) from “CartoDB Basemaps” (https://github.com/CartoDB/CartoDB-basemaps) designed by Stamen and Paul Norman for CartoDB Inc. Basemap of East New Britain province from Esri World Street Map (sources: Esri, DeLorme, HERE, USGS, Intermap, iPC, NRCAN, Esri Japan, METI, Esri China (Hong Kong), Esri (Thailand), MapmyIndia, Tomtom) was obtained from https://leaflet-extras.github.io/leaflet-providers/preview/#filter=Esri.WorldStreetMap). Both maps are licensed under CC-BY 4.0.

East New Britain Province (ENBP, population approximately 376,566) comprises the eastern half of the island of New Britain in PNG (5°10′ 0ʺ S, 151° 45′ 0ʺ E). It is divided into four districts: Gazelle, Pomio, Kokopo, and Rabaul (Fig 1 and Table 1). Most people reside in the northeastern part of the province. Cocoa, copra, and palm oil are the major cash crops. Rabaul and Kokopo districts are more urbanized, with more modern structures (housing with window screens), easy access to paved roads, and electricity. Gazelle and Pomio are rural districts with less infrastructure.

**Table 1:** Baseline and one year post MDA-IDA impact survey characteristics.

*Survey time*	*Cluster type*	*Age category (years)*	*No. sampled*	*Mean Age ± SD (Range)*
Baseline	Random N = 30	Children (6–9)	1,121	7.4 ± 1.2 (6–9)
		Others (≥ 10 years)	1,449	29.6 ± 14.4 (10–77)
	Purposive N = 19	Children (6–9 )	785	7.4 ± 1.2 (6–9 )
		Others (≥ 10 years)	906	29.9 ± 14.3 (10–72)
	All sites N = 49	Children (6–9 )	1,906	7.4 ± 1.1 (6–9 )
		Others (≥ 10 years)	2,346	29.6 ± 14.4) (10–77)
One year Post–MDA	Repeat villages from Baseline N = 24	Children (6–9 )	416	7.6 ± 1.2 (6–9 )
		Others (≥ 18 years)	2,016	34.8 ± 14.1 (10–88)
	Geo–spatial Selection N = 23	Children (6–9 )	278	7.6 ± 1.3 (6–9 )
		Others (≥ 10 years)	1,901	34.7 ± 13.5 (18–81)
	All sites N = 47	Children (6–9 )	694	7.6 ± 1.2 (6–9 )
		Others (≥ 10 years)	3,917	34.8 ± 13.8 (10–88)

### Mass drug administration

In November 2019, MDA with IDA (MDA-IDA) was conducted throughout the province. Before MDA, community health workers (CHW) were essential in mobilizing the community for MDA through meetings (known as Toksave) and training community distributors. MDA was distributed over three weeks to facilitate travel to remote areas, better capture mobile populations, and provide time for mop-up. The whole MDA process was coordinated by a highly engaged LF/NTD task force in the province that involved community leaders and other stakeholders, ensuring campaign success.

### Study design and village selection

The village is the primary sampling unit. We adopted three sampling methodologies to assess the LF prevalence and evaluate the impact of IDA in ENBP. At baseline, we used i) population proportionate (PPS) sampling to assess the population mean prevalence (N = 30) and ii) surveyed additional villages suspected or known to have a high prevalence of LF (N = 19). Post-MDA, we re-sampled villages that were identified to have CFA >2% and/or >1% Mf pre-MDA. We used a geostatistical sampling design to identify additional sites post-MDA at high risk for failing to reach the <1% Mf threshold following MDA. Because LF infection prevalence was low in children, we only sampled children 6–9 in villages where we observed LF infection at baseline, otherwise we only sampled adults 18 years and older.

Since the *Anopheles* mosquitos transmit LF in PNG, the sample size was powered to detect a 2% CFA threshold for each of two groups; i) children aged 6–9 years old and other community members ≥10 years, and ii) during the post-MDA survey children aged 6–9 years old and other community members ages ≥18 years. The post-MDA target population and sample size were adjusted based on WHO interim guidelines of a 1% Mf threshold in adults ≥18 years, based on the upper 1-sided 95% confidence interval to assess the impact of IDA (IDA Technical Report 2022). The selected households within each village was adapted from the WHO guidelines for evaluating coverage surveys and monitoring [18]. This approach divided the villages into segments with approximately an equal number of households and a segment. A segment was randomly selected and surveyed. Given this sampling frame, we wanted an equal likelihood of sampling all 6–9-year-old children and ≥10 years present per household. Fifty households in a segment were selected to reach 100 individuals per village [19]. We also considered that there may be households without 6–9-year-olds and expanded our sampling to include >50 households if we did not achieve the required number of children with adults. These households were randomly selected from a list of household numbers.

At baseline, we surveyed all members of 49 randomly selected households to find children aged 6–9 years and individuals aged 10 and above, until we reached 100 participants (approximately 50 children and 50 adults aged 10 and above) per village. If a randomly selected household consisted of only adults, it was excluded.

One year post-MDA, we examined adults (18 years and above) in 23 newly selected villages using model-based geostatistics MBG. In the 24 villages examined before and after MDA (with more than 2% CFA at baseline), we surveyed approximately 50 children and 50 adults in the villages with CFA positivity in 6–9-year-old children identified at baseline. Only adults were sampled post-MDA (approximately 100) in villages without CFA positivity in children. All eligible individuals in the household were examined.

### Socio-economic assessment

At baseline, we adapted a socioeconomic impact (SEI) score adapted from prior studies [20,21] for each village by assessing access to potable water, the use of window screens, and the level of education for each household. In calculating the SEI scores, we counted 1 point if a household had access to a water tank or pipe (0 for river/stream or public pump/well) and 1 point if window screens were used (0 for no screens). In scoring educational status, each adult (18 years or older) received a score based on their highest level of education attained: 3 points for college or vocational training, 2 points for high school graduates, 1 point for an 8th-grade education, and a score of 0 if the adult had not attended school through the 8th-grade. The composite educational score was computed by taking the average score of each adult in the household. The total SEI score for each household was obtained by adding the educational average to the water and screen scores. To get the SEI score for each village, we averaged all the households in that village. Higher SEI scores indicate higher socioeconomic status. We also computed 95% confidence intervals and ranges for village SEI scores. The SEI scores were used in the geostatistical model for sampling at 1-year post-MDA.

### Coverage survey

A cluster sampling approach was applied to determine drug coverage after one round of MDA [18]. The primary sampling unit was local level government (LLG) within each of the four districts, and each LLG contained wards that consisted of many villages. The Rotary Against Malaria (RAM) survey for 2016 was used as the denominator of the village population. In this survey, 8 LLGs or clusters in each of the four districts were selected by population proportionate sampling (PPS), which were: Gazelle Central Rural (Gazelle), Lasul Baining Rural (Gazelle), Vunadirir Toma Rural (Gazelle), Bitapaka Rural (Kokopo), Kokopo Vunamami Urban (Kokopo), East Pomio Rural (Pomio), Balanataman Rural (Rabaul) and Rabaul Urban (Rabaul). Gazelle Central Rural LLG was divided into three independent clusters because of the large population size relative to the rest of the LLGs in ENBP. Population proportionate sampling then selected the villages within the LLGs for the coverage surveys. Across the LLGs, 45 villages were surveyed for drug coverage. Twenty-five of the 45 villages were in the Gazelle district, 10 in Kokopo, 5 in Pomio, and 5 in Rabaul. Of the 45 villages surveyed, 15 (33.3%) were included in the baseline LF prevalence survey.

### Procedures

Fingerstick blood samples were collected during the surveys for CFA testing with Filariasis Test Strips (FTS) (Alere, Scarborough, ME, USA) according to the manufacturer’s protocol. Tests with no control line were invalid and repeated. The FTS detects a biomarker for infection with *W. bancrofti* (Wb) adult worms, and it has a high sensitivity for detecting persons with microfilaremia. In community studies in PNG, all Mf-positive individuals had positive FTS results [22]. In this study, persons with positive FTS results were tested for Mf with 60-μl thick blood smears prepared from fingerstick samples collected between 9 PM and 1 AM, as previously described [22]. Two microscopists reviewed the slides. If Mf counts were highly discordant or one reader identified Mf and the other did not, a third reader reviewed the slide.

### Data acquisition and management

The study used Epi Info (Centers for Disease Control) software to capture and transfer data to a REDCap database (v11.0.3, Vanderbilt University). The data were entered by trained members of the PNG research team on the day of enrollment. A participant key (separate from the REDCap database and maintained at PNG Institute for Medical Research) linked study ID numbers with personal identifying information, such as name and date of birth. The participant key was not shared with Case Western Reserve University investigators or staff.

### Geostatistical and statistical analysis

The geostatistical methods sampled villages one-year post-MDA-IDA following adaptive geostatistical designs [23]. The methodological framework includes prevalence data modeled to describe the number of people infected with a particular disease or outcome at a given location in a specific region of interest with a vector of associated covariates. The model accounted for the spatial correlation between villages based on their proximity in space, whereby closer villages were assumed to be more correlated than villages further apart. The current analysis also modeled the association between locations on distinct land masses through the Euclidean distance - i.e., the ordinary “straight-line” distance. The predictions were carried out on a 1 by 1 km regular grid. Grid locations whose distance from the closest data locations was larger than 15 km were excluded from the predictions. There were 748 villages in ENBP, of which we selected 49 that had the probability of exceeding the 2% CFA prevalence threshold. This was accomplished as follows:

The geostatistical model selects the number of positively tested cases for LF,  *Y*_*i*_, out of *n*_*i*_ sampled individuals at the i-th village location *x*_*i*_. We then assume that conditionally on spatial Gaussian process $S(x_{i}),$ the *Y*_*i*_ follow a set of mutually independent Binomial variables with logit-link function. Let $p(x_{i})$ denote the probability of a positive test for an individual living in a village at location *x*_*i*_. The linear predictor is then expressed as


$$\log\left\{\frac{p\left(x_{i}\right)\;}{1-p\left(x_{i}\right)\;}\right\}=\;\beta_{0}+\beta_{1}d_{1}\left(x_{i}\right)+\beta_{2}d_{2}\left(x_{i}\right)+S\left(x_{i}\right)$$

where: $d_{1}(x_{i})$ is a covariate which quantifies the environmental exposure to LF at location *x*_*i*_ by combining information on relative humidity, annual precipitation, annual temperature, elevation and distance from the sea using principal component analysis; $d_{2}(x_{i})$ is the socio-economic impact (SEI) score. The rationale for the inclusion of $S(x_{i})$ is to account for unmeasured risk factors that, in addition to $d_{1}(x_{i})$ and $d_{2}(x_{i}),$ affect LF prevalence. We assume $S(x_{i})$ to be an isotropic and stationary Gaussian process with exponential covariance function given by


$$Cov\left\{S\left(x_{i}\right),S\left(x_{j}\right)\right\}=\sigma^{2}exp\left\{-\left|\left|x_{i}-x_{j}\right|\right|/\varphi \right\}$$

where *σ*^2^ is the variance of the spatial process $S(x_{i}),$ $\left|\left|x_{i}-x_{j}\right|\right|$ is the distance, in km, between location *x*_*i*_ and *x*_*j*_ and *φ* is a scale parameter which regulates how fast the spatial correlation decays to zero for increasing distance $\left|\left|x_{i}-x_{j}\right|\right|$

We fitted the model given in the above equation using Monte Carlo maximum likelihood methods included in the PrevMap package [24].

After fitting the geostatistical model described above, we obtained the predictive probability of exceeding 2% prevalence thresholds (or EP_2%_) at each of the 49 sampled and 699 unsampled villages. Interpreting the exceedance probabilities EP_2%_ is: values of EP_2%_ close to 0 indicate a high probability that prevalence is below 2%; values of EP_2%_ close to 1 indicate a high probability that prevalence is above 2%; and, if values of EP_2%_ are close to 50%, this corresponds to the highest level of uncertainty, with an equal probability of exceeding or not the 2% threshold. To identify locations that were more likely to have a higher than 2% prevalence baseline, we proceed through the following iterative steps: i) Set the minimum value of EP_2%_ say α, used to include new villages in the sample to 95%, ii) among the 49 sampled villages select those for which EP_2%_> α. If a Ward is represented by more than one village, take only the village with the highest EP_2%_. Denote this final set of villages as S_1_, iii) among the 699 sampled villages, select those for which EP2%> α. If a Ward is represented by more than one village, take only the village with the highest EP2%. Denote this final set of villages as S_2_, iv) if the sum of the size of S_1_ and S_2_ exceeds 50, then increase α to αnew= α+1/100; if the sum of the size of S_1_ and S_2_ is below 50, then decrease α to αnew= α-1/100; repeat steps 1 to 3, v) if the sum of the size of S_1_ and S_2_ is 50, then stop with S_1_ and S_2_ corresponding to the set of villages to be sampled. A sample size of 50 villages was selected as the upper limit in the model, which is comparable to the number of villages surveyed at baseline.

Descriptive statistics were calculated as frequencies and proportions for categorical variables ± 95% confidence intervals. Median and interquartile ranges, or mean ± SD, were used for demographic variables. Comparisons between treatment groups for demographic and infection variables were performed using chi-square and Fisher’s exact tests (as appropriate) for categorical variables using SAS (v 9.4, SAS Institute).

### Mapping software

The geostatistical probability and prevalence maps were developed using R statistical computing software (v 4.4.1) [25] with PrevMap [24] and leaflet [26] packages. The ENBP boundaries for the geostatistical map was from DIVA-GIS [27]. Grid for model outputs were generated on the boundaries and plot of the raster was created of the model data. The map was created on a blank background and no basemap. The ENBP boundaries for the CFA and Mf prevalence maps were from the global database of administrative boundaries [28].

## Results

The evaluation unit in PNG is the province, although the districts better capture the province’s geographic and social heterogeneity. Overall, 4,252 participants were enrolled for the baseline survey and 4,611 for the post-MDA-IDA survey. Table 1 describes the population characteristics and sampling design pre- and post-MDA-IDA.

### Baseline LF infection parameters

To estimate the mean LF prevalence in ENBP, we used the WHO-recommended PPS sampling of 30 villages (Table 2). This corresponded to 8 villages in the Kokopo District, 11 in the Gazelle District, 5 in the Pomio District and 3 in the Rabaul District which corresponds to districts from the highest population (Gazelle) to the lowest (Rabaul). The estimated mean LF prevalence was low, with CFA prevalence of 2.3% (95% CI 1.8–2.9%; 59/2,561) and Mf prevalence of 0.6% (95% CI 0.3–0.9%; 14/2,561). In children 6–9 years of age only 0.7% (95% CI 0.3–1.4; 8/1,121) were CFA positive, and none were Mf positive. However, LF infection was highly heterogeneous. Eight of 30 (26.7%) PPS-selected villages had CFA >2%.

**Table 2:** LF Infection Parameters in PPS selected Villages pre-MDA.

	Total CFA and MF % in randomized selected villages by PPS					6–9 years					≥10 years				
District	Population	CFA+ (N)	CFA+% (95% CI)	Mf+ (n)	Mf+% (95% CI	N	CFA+ (N)	CFA+% (95% CI)	Mf+ (N)	Mf+% (95% CI)	N	CFA+ (N)	CFA % (95% CI)	Mf+ (N)	Mf % (95% CI)
**Kokopo**	691	6	0.87 (0.3–1.9)	1	0.1 (0.0–0.8)	340	1	0.3 (0.0–1.6)	0	–	351	5	1.4 (0.5–3.3)	1	0.3 (0.0–1.6)
**Gazelle**	1072	16	1.5 (0.9–2.4)	5	0.5 (0.2–1.1)	451	5	1.1 (0.4–2.6)	0	–	621	11	1.8 (0.9–3.2)	5	0.8 (0.3–1.9)
**Pomio**	398	33	8.29 (5.8–11.5)	7	1.8 (0.7–3.6)	145	2	1.4 (0.2–4.9)	0	–	253	31	12.3 (8.5–16.9)	7	2.8 (1.1–5.6)
**Rabaul**	400	4	1.0 (0.3–2.5)	1	0.3 (0.0–1.4)	185	0	–	0	–	215	4	1.9 (0.5–4.7)	1	0.5 (0.0–2.6)
**Total**	**2561**	**59**	**2.3 (1.8–2.9)**	**14**	**0.6 (0.3–0.9)**	**1121**	**8**	**0.7 (0.3–1.4)**	**0**	**–**	**1440**	**51**	**3.5 (2.7–4.6)**	**14**	**0.9 (0.5–1.6)**

The CFA prevalence of <2.0% among 6–9-year-olds would suggest that ENBP might not have ongoing LF transmission based on the WHO transmission assessment survey (TAS) of lower primary school-aged children 6–7 years of age using a 2% CFA threshold. To examine this age group, we stratified the 1,906 6–9 year-old children sampled into those 6–7 years of age (N = 1,075), representing 54% of children examined. Thus, there would be approximately 24,000 6–7 year of age children in ENBP (based on the population pyramid of PNG [populationpyramid.net] of 5–9 year olds approximately 45,188 × 0.54 = 24,400). Using a community-based TAS algorithm of a population size of ~20,000, the sample size would be ~1,500 in 30 clusters, like the current baseline survey. The CFA prevalence in 6–7-year-olds was 0.6% (4/625), below the 2% CFA threshold; although this sample size is smaller than the WHO recommendation, the upper 95% CI is <2%. Thus, using the standard TAS survey might have led to an erroneous conclusion that ENBP would not need MDA. The problem with PPS was sampling the whole province as the evaluation unit, that failed to capture the high heterogeneity of LF infection.

**Fig 2:**
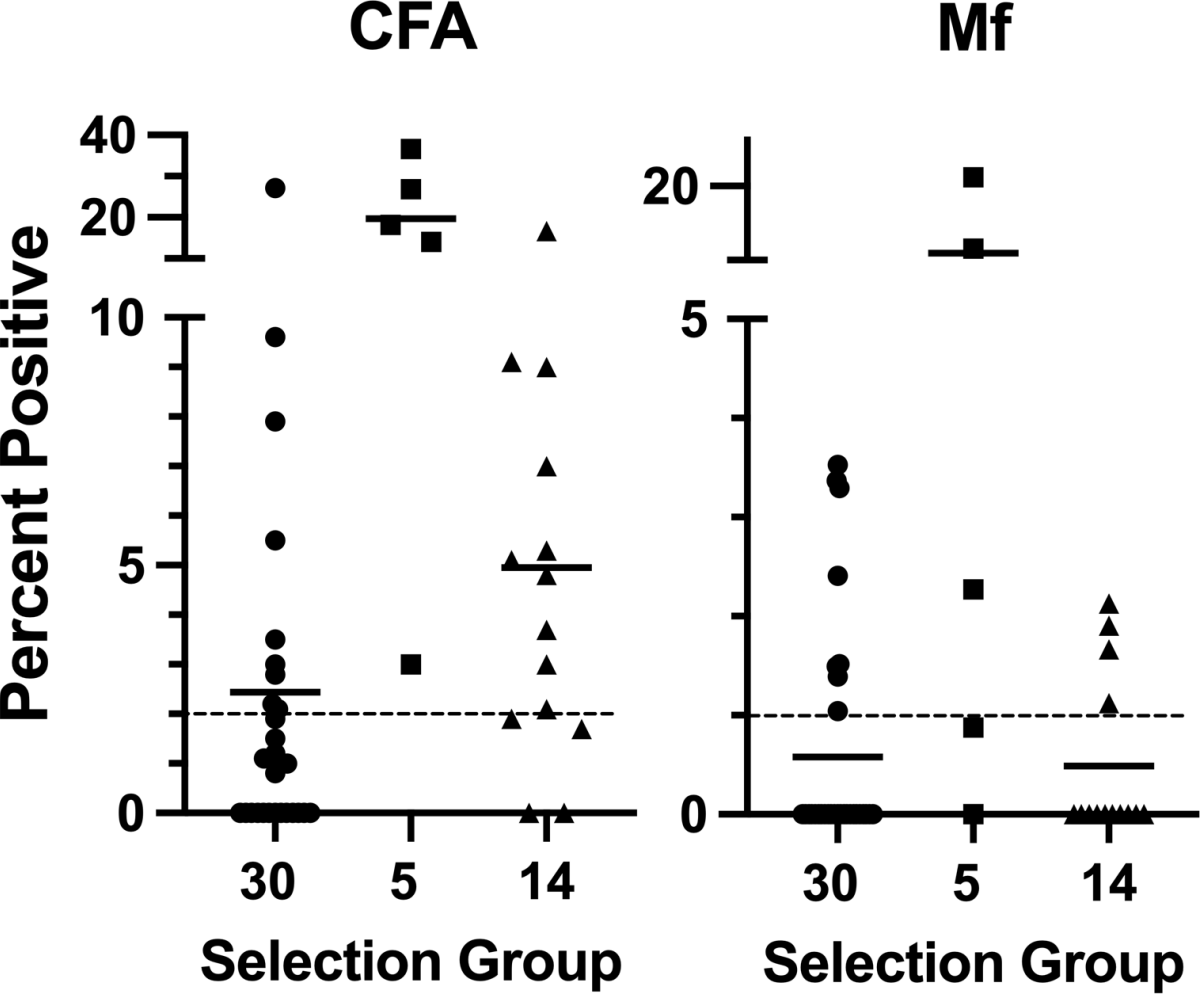
LF infection parameters in villages according to different sampling strategies at baseline. Thirty villages used a population proportion random sample. ENBP Provincial Health Authority purposefully selected five additional villages with prior evidence of LF. Fourteen more villages were chosen to adequately sample rural areas in Pomio (N = 9) and Gazelle (N = 5) districts. The dashed line on the left panel and the red line on the right panel represent WHO-specified thresholds of 2% CFA and 1% MF rates, suggesting ongoing LF transmission in villages above these thresholds.

**Fig 3:**
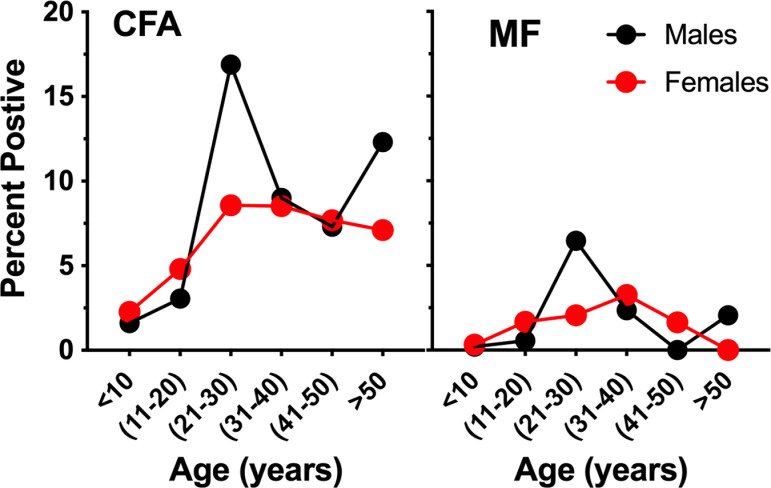
Circulating filarial antigen (CFA) and microfilaremia (MF) prevalence by age groups pre-MDA for 49 villages.

The purposeful sampling of five villages with previously known LF infection and 14 additional villages suspected of infection highlighted the focal nature of LF and identified villages with higher LF prevalence (Table 3). The five purposively selected villages known to have LF all had >2% prevalence of CFA, and 3 had Mf prevalences of >1% (Fig 2). Of the additional 14 purposely selected villages in rural villages, 10 (71.4%) villages had ≥2% CFA, and 4 of 14 (28.6%) had >1% Mf prevalence. Overall, we sampled 4,252 participants in 49 villages with 1,906 children aged 6-9 years, and 2,346 were individuals aged ≥10 years (**Table A in** S2 Table). Females accounted for 48% of the 6–9 years sampled and 61% of those ≥10 years. Circulating filarial antigen and Mf prevalence increased with age, with the highest prevalence in men between 21 and 30 (Fig 3). Combining all groups, twenty-four (48.9%) of the 49 villages had ≥2% CFA, and 14 met the ≥1% Mf threshold (Fig 2 **and** S1 Table)

**Table 3:** LF Infection Parameters in Purposively Selected Village pre-MDA.

	Total CFA and MF % in purposive selected villages					6–9 years					≥10 years				
District	N	CFA+ (N)	CFA+% (95% CI)	Mf+ (N)	Mf+% (95% CI	N	CFA+ (N)	CFA+ % (95% CI)	Mf+ (N)	Mf+ % (95% CI)	N	CFA+ (N)	CFA+% (95% CI)	Mf+ (N)	Mf+% (95% CI)
Kokopo	266	63	23.7 (18.7–29.3)	28	10.5 (7.1–14.8)	140	22	15.7 (10.1–22.8)	5	3.6 (1.2–8.1)	126	41	32.5 (24.5–41.5)	23	18.3 (11.9–26.1)
Gazelle	415	24	5.8 (3.7–8.5)	1	0.24 (0.0–1.3)	209	5	2.4 (0.8–5.5)	0	–	206	19	9.2 (5.6–14.0)	1	0.5 (0.0–2.7)
Pomio	1010	67	6.6 (5.2–8.4)	9	0.9 (0.4–1.7)	436	2	0.5 (0.1–1.7)	0	–	574	65	11.3 (8.9–14.2)	9	1.6 (0.7–2.9)
Rabaul*	0	0	–	0	–	–	–	–	–		–			–	
**Total**	**1691**	**154**	**9.1 (7.8–10.6)**	**38**	**2.3 (1.6–3.1)**	**785**	**29**	**3.7 (2.5–5.3)**	**5**	**0.6 (0.2–1.5)**	**906**	**125**	**13.8 (11.6–16.2)**	**33**	**3.6 (2.5–5.1)**

*Villages in the Rabaul district were not purposively selected because they were categorized as low-risk, having <2% CFA and Mf prevalence in previously surveyed villages.

**Fig 4:**
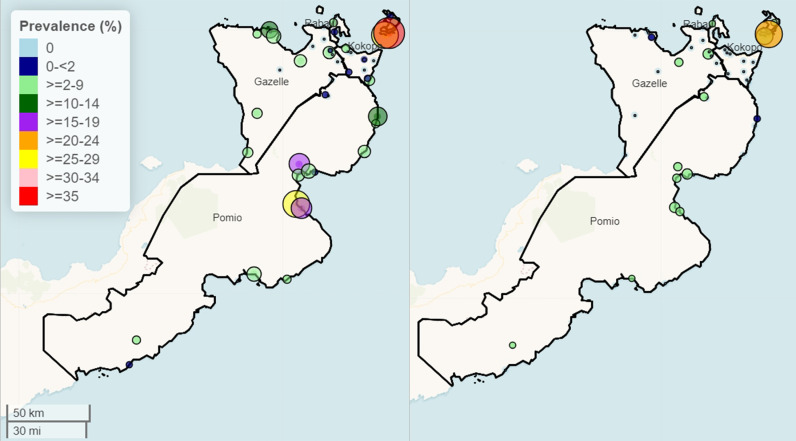
Spatial distribution of lymphatic filariasis circulating antigen positivity (CFA, left panel) and microfilaremia rates (Mf, right panel) throughout ENBP before mass drug distribution. Small solid dots indicated communities without any detectable LF infection. The colors and circle size represent LF prevalence for CFA or Mf. Mapping: The map was made using R package, leaflet. Map data copyright from OpenStreetMap contributors and data is available under the Open Database License at https://www.openstreetmap.org/copyright. Basemap from “CartoDB Basemaps” (https://github.com/CartoDB/CartoDB-basemaps) designed by Stamen and Paul Norman for CartoDB Inc., licensed under CC-BY 4.0.

The identified villages with the highest prevalence concentrated in the Duke of York Islands (DOY) in the Kokopo district and Wide Bay Area along the eastern coast of the Pomio District (Fig 4). Gazelle and Rabaul Districts had lower LF infection prevalence.

### Mass drug administration

The ENBP Provincial Health Authority administered the first round of MDA in November 2019 to 307,568 of 376,566 total residents of ENBP (81.7% reported or administrative coverage, S6 Table). The reported coverage varied by district, Pomio (72.8%) and Gazelle (83.8%), rural districts, to 93.3% and 96.7% in the more urban districts of Kokopo and Rabaul, respectively. Of note, only DEC plus Albendazole was administered to children 2 to 4 years of age. In December 2019, an independent coverage survey was performed to validate the administrative coverage survey. This included 2,598 individuals in 450 randomly selected households in 45 randomly selected villages. Over 80% of the individuals reported taking treatment in all age groups except those >50 years of age (S1 Fig).

### Model-based geostatistical sampling post-MDA LF infection parameters

**Fig 5:**
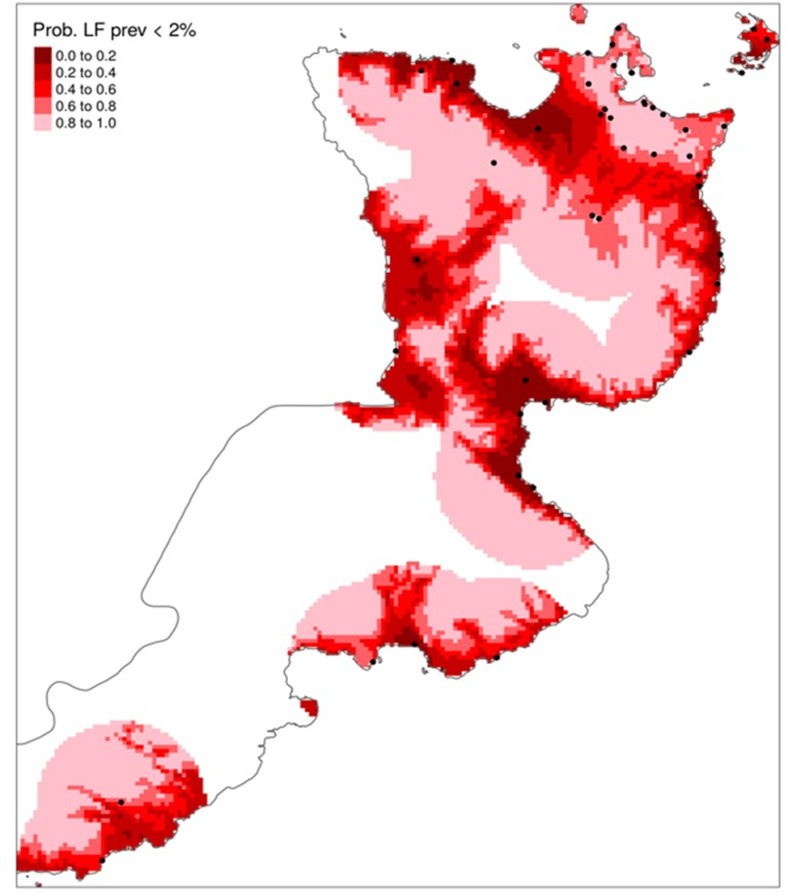
Geostatistical probability map of LF prevalence. The dark red area has a low probability that CFA prevalence is <2%; thus, these areas are more likely to have LF. The map resolution is 1 km. White regions have insufficient data to assess LF risk. Risk mapping was generated based on covariates of elevation, rainfall, temperature, distance from the sea, socioeconomic status, and LF prevalence using baseline survey villages (black dots). Mapping: Map was developed using R package, PrevMap. No basemap was used in the creation of this image.

Because the pre-MDA PPS oversampled urban areas with lower LF prevalence and under-sampled rural areas with higher LF prevalence, we undertook a sampling strategy to reflect better the geographical distribution of LF in ENBP. Thus, we used model-based geostatistical (MBG) as an alternative method to select villages for monitoring and evaluation during the post-MDA 1-year follow-up survey. The model incorporated baseline CFA prevalence data and covariates associated with the probability of LF infection: relative humidity (RH), annual precipitation (AP), annual temperature (AT), elevation (E), distance from the sea (DS), socio-economic status (SEI) and malaria prevalence. Because of the small sample size, we combined the covariates using principal component analysis (PCA) and used the first component (PCA1) as a predictor for LF. The sample size was too small to establish which variables were most informative for predicting areas with >2% CFA. The model selected the 24 villages at baseline with >2% CFA positivity (as expected) and 23 additional villages across the four districts of ENBP that were likely to have >2% CFA positivity (Fig 5).

**Fig 6:**
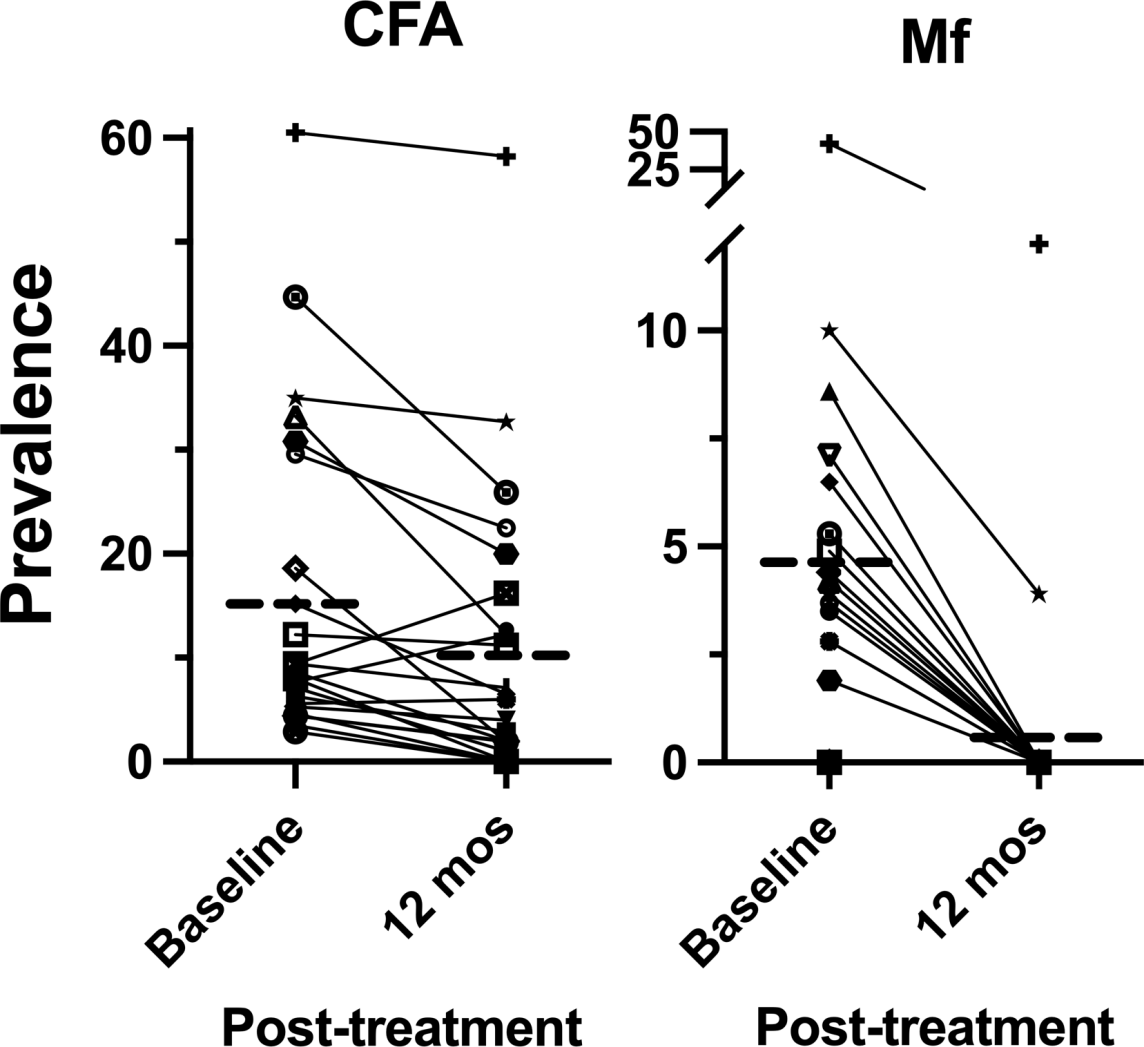
Impact of treatment on CFA and Mf prevalence in 24 villages before and after MDA. Prevalence estimates are for ≥18 years old with N = 816 before MDA and N = 2015 12 months post-MDA. Dashed lines represent means.

Following MDA-IDA, a total of 4,611 participants were sampled in the 47 villages. Twenty of 23 (87%) newly selected villages had >2% CFA prevalence, indicating an efficient selection of communities using MBG. Children were surveyed in 19 of the 47 villages, and adults (≥18 years) were studied in all 47 (Table 4). Fig 6 shows LF infection parameters pre- and post-MDA, for age-matched adults (≥18 years) in 24 of 47 villages monitored at baseline and 1 year post-MDA. The CFA mean prevalence decreased by 38.6% from 17.3% (141 of 816) to 10.6% (215 of 2,015). By contrast, Mf prevalence decreased by 90.1% from 4.9% (40 of 816) to 0.45% (9 of 2016). Eleven of the 24 sentinel sites also surveyed children post-MDA. Comparison of LF infection parameters in these 11 villages in children showed a decrease in CFA prevalence by 30.1% from 7.2% (30 of 417) to 4.9% (18 of 365) and completely cleared Mf infection in children; from 1.2% (5 of 417) to 0% (0 of 365).

**Table 4:** Study population demographic and infection characteristics of 6–9 and ≥18 years at one-year post-MDA.

*District*	*Villages surveyed*	*N*	*Percent Females*	*CFA*+ *(N)*	*CFA+ (95%CI) [range]*	*Mf*+ *(N)*	*Mf +*	*Villages ≥2% CFA+*	*Villages ≥1% Mf+*
**6–9 years**									
Kokopo	5	**231**	50.7	9	3.9 (1.8–7.3) [0–10.6]	0	–	2	0
Gazelle	2	**93**	52.9	1	1.1 (0.0–5.9)	0	–	1	0
Pomio	12	**370**	46.6	10	2.7 (1.3–4.9) [0–6.2]	0	–	5	0
Rabaul	0	**0**		–	–		–	–	–
**Total**	**19**	**694**	**48.8**	**20**	**2.9 (1.8-4.4) [0-10.6]**	**0**		**8**	**0**
**≥18 years**									
Kokopo	9	**627**	64.9	91	14.5 (11.9–17.6) [0–58.2]	10	1.6 (0.8–2.9) [0–12.7]	8	3
Gazelle	12	**1,107**	54.2	34	3.1 (2.1–4.3) [0–18.7]	0	0.1 (0.0–0.1) [0.9]	9	0
Pomio	25	**2,083**	55.8	227	10.9 (9.6–12.4) [0–28.4]	5	0.3 (0.1–0.5) [0.9–1.0]	22	3
Rabaul	1	**100**	57.6	0	0	0	0	0	0
**Total**	**47**	**3,917**	**56.8**	**352**	**9.0 (8.1–10.0) [0–58.2]**	**15**	**0.4 (0.2–0.6) [0–12.7]**	**39**	**6**

Importantly, in the 23 villages selected by MBG, we identified one Mf-positive individual in each of the four villages. The four villages were <1% Mf cutoff using the lower 95% confidence interval based on each village’s total population of adults.

**Fig 7:**
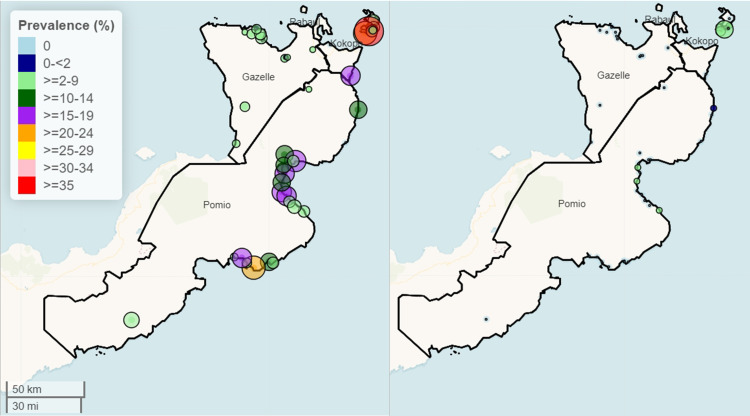
The Geographical distribution and prevalence of villages were sampled following MDA. The left panel shows CFA prevalence, and the right panel shows Mf prevalence. Small white dots are villages without detectable CFA or Mf in participants. The color gradient of the maps corresponds to the different CFA prevalence (left panel). The size of green circles correlates with Mf prevalence, with the smallest green circles being 1% Mf. Mapping: The map was made using R package, leaflet. Map data copyright from OpenStreetMap contributors and data is available under the Open Database License at https://www.openstreetmap.org/copyright. Basemap from “CartoDB Basemaps” (https://github.com/CartoDB/CartoDB-basemaps) designed by Stamen and Paul Norman for CartoDB Inc., licensed under CC-BY 4.0.

Concerning all 47 villages surveyed post-MDA, Mf prevalence decreased to 0.38% (15 of 3,917). Two villages in Duke of York Islands (Figs 6 and 7) exceeded >1% Mf with 95% lower confidence interval. CFA prevalence of 9.0% (352 of 3,917; post-MDA-IDA, S3 Table) was comparable to the baseline CFA prevalence of 9.6% (151 of 1,581; **Table E in** S2 Table). There are limitations to a direct comparison of the 49 villages pre-MDA with 47 post-MDA because of differences in sampling design, e.g., primarily adults in post-MDA.

Fig 7 shows the CFA and Mf prevalences in sampled villages post-MDA. Two districts, Rabaul and Gazelle, had no sentinel villages with ≥1% Mf positivity. However, six villages had 1 or more LF-positive individuals, three in the Duke of York Islands (Kabatira, Karawara, and Utuan, 1%, 2%, and 7%, LF prevalences respectively, and the others were clustered along the East Coast in the Wide Bay area of the Pomio district. Only two villages, Utuan and Karawara, would exceed >1% Mf prevalence based on lower 95% confidence interval.

## Discussion

East New Britain was the first province in PNG to implement MDA with IDA. The program was highly successful and achieved >80% drug coverage after the first round of MDA. Following one round of MDA, overall Mf prevalence decreased from 1.2% pre-MDA to 0.4%, well below the WHO pre-TAS Mf prevalence target of <1%. However, some areas with high prevalence at baseline failed to reach this target. One of the four districts in the province detected no Mf-positive individuals, and another district detected one Mf-positive individual following MDA-IDA, suggesting little risk of ongoing LF transmission. By contrast, 6 of 47 villages surveyed had 1 or more Mf-positive individuals post-MDA. Only two of these villages can be classified ≥1% Mf positivity based on WHO criteria of lower 95% confidence interval. These remaining Mf-positive communities were clustered in small geographic areas with high LF prevalence pre-MDA.

Current WHO guidelines recommend another round of MDA for the whole province [14], which has the advantage of treating other parasitic infections or capturing Mf carriers from nearby unsampled villages. This study highlights the strengths and weaknesses of different sampling strategies and endpoints to assess LF prevalence where infections are highly focal and prior information on LF is limited. The population proportional sampling strategy (PPS, WHO recommended) used at baseline underestimated the LF burden. This is because PPS oversampled communities in urban and peri-urban areas with little LF and under sampled communities in rural areas with more LF. This sampling strategy may not be appropriate in EUs that are highly heterogeneous for LF, as observed in ENBP and other PNG provinces. However, it may be applicable in smaller, more homogenous areas with LF infection or rural areas. Still, it increases the number of villages to be sampled and the cost. Results from this study also add to recent reports that highlight the limitations for TAS of young school-aged children for post-MDA stopping decisions. Before MDA, only 0.7% of children 6–9 years of age in ENBP were CFA positive, and 0.6% among children 6-7 years old, the TAS target population, which is well below the 2% threshold believed to suggest recent or ongoing LF transmission. Indeed, ENBP probably could have passed TAS performed according to WHO protocols before any MDA, even though the province had areas with high endemicity at that time. Since only a few infections in young children were detected before MDA, TAS would have provided unreliable information for MDA-stopping decisions following MDA. Children may not be suitable sentinels for assessing LF transmission as their exposure often differs from adults. Despite low infection prevalence in children, ENBP was known to be endemic for LF based on prior surveys conducted by health officials and confirmed by baseline surveys reported here. However, many other areas in PNG do not have detailed information on LF prevalence because LF testing and reporting are not included in the country’s National Health Information Surveillance System. Our results from ENBP suggest that PPS and TAS may not be the best way forward for LF elimination efforts in other regions in PNG.

This study reinforced the difficulty of using CFA alone as an endpoint for assessing the impact of IDA on LF transmission. One round of MDA reduced CFA prevalence by 30.1% in children 6–9 years of age and 38.6% in adults; this lower CFA reduction has been observed in prior clinical trials [10,11,13–15]. However, worms that survive IDA treatment can continue to release CFA for years after Mf clearance [11]. One approach to this problem for MDA-stopping decisions and post-MDA surveillance is to use CFA as a screening test in adults, with Mf testing restricted to those with positive antigen tests. It should be safe to stop MDA if Mf prevalence has been reduced to very low levels in adults despite a CFA prevalence of >2%. Mosquitoes cannot transmit LF without access to Mf.

We used geostatistical modeling to more efficiently identify communities at risk for LF and assess the impact of one round of MDA. Geostatistical modeling is increasingly used for mapping tropical diseases in low-resource settings where disease data are limited or insufficient information is available to provide insight into the infection intensity, distribution, affected populations, and environmental and seasonal patterns [29]. It incorporates information on known environmental and sociodemographic risk factors for LF, including elevation, vegetation, and population density. It has been increasingly used to map tropical diseases, such as malaria, Chagas disease, LF, and trachoma, where data are limited or nonexistent [30–33]. The method has also been applied to predict residual LF hotspots post-MDA. Our results show that geostatistical modeling was more helpful than PPS for identifying communities with high CFA post-MDA using baseline infection data and environmental variables. This approach should help inform programmatic decisions [26] by facilitating the detection of persistent infections. East New Britain province is highly heterogeneous for LF, with low infections predominantly found in areas closer to urban areas or main roads, compared to remote, logistically challenging, and inaccessible locations. The PPS had limitations in adequately identifying high-risk locations in ENBP because the whole province was considered the EU and failed to account for highly heterogeneous risk among districts. The districts could be EUs, and PPS could be used for each district. However, the geostatistical sampling approach was more efficient in sampling sites likely to exceed the LF threshold. The advantage of the geostatistical modeling approach is the versatility of using available LF infection data and other covariates to predict high-risk locations for sampling. Alternatively, the size of the EU can be reduced after the first MDA-IDA to target localities with high LF, as we showed for the Duke of York Islands, Kokopo district. We show that site selection using a geostatistical model is more efficient than PPS because it accommodates infection and environmental variables predictive of LF. Therefore, geostatistical model selected sites can be used to determine the decision to stop MDA in an EU. The sampling strategy can be cost-effective in low- and middle-income countries where the distribution of LF is heterogeneous. The PPS is more reliable in locations homogenous for LF. The limitations of geostatistical modeling are that it has yet to be widely available, it takes technical expertise to run the programs, and because of its limited use so far, there remains insufficient data to better identify individual factors in the model most predictive of LF infection.

The previous recommendations from WHO, stating that decisions to stop mass drug administration (MDA) should be based on the mean prevalence in an evaluation unit (EU), are inadequate in areas like PNG, where LF distribution is highly heterogeneous. As lymphatic filariasis (LF) transmission levels approach elimination, the remaining LF infections become increasingly concentrated in certain areas, and these areas should be targeted for potential further MDA interventions. This study emphasizes this concept. One round of MDA with high coverage likely halted LF transmission in two of the four districts in East New Britain Province (ENBP) with low initial LF prevalence. Additional rounds of MDA could focus on the two districts with clusters having over 1% microfilaria (Mf) prevalence or specific clusters within the district, such as the Duke of York Islands. The WHO recommends two rounds of MDA, which may reach those who missed the first round and address other infections. However, in LF-endemic areas like PNG, where LF infection may be highly localized, conducting a single round of MDA achieving over 80% coverage could be adequate, and an impact survey could take place after the first round of MDA. This approach would enable additional rounds of MDA to target areas with over 1% Mf prevalence.

There are some limitations to this study. Before MDA, we sampled many children so that the median age of our sample was 12 years. In contrast, our post-MDA survey focused more on adults (median age of 29 years). Since LF is age-dependent, a direct comparison of infection prevalence before and after MDA required a comparison of an age-matched subset of individuals sampled before and after MDA. Another limitation of the study is that it only assessed the impact of one round of MDA, although WHO recommends two rounds. This was intentional because we wanted to determine whether a single round of MDA with high coverage would reduce Mf prevalence to <1%. This did occur in many areas, but hotspots remained.

In summary, this study showed that a single round of MDA-IDA significantly reduced the prevalence of Mf to very low levels in most endemic areas in ENBP. This success was due to the excellent efficacy of IDA in clearing Mf in PNG and the high MDA coverage provided by public health officials in ENBP. Further surveys will be needed to determine if a second round of MDA-IDA will eliminate any remaining infection hotspots. The findings of this study will support the broader use of IDA for LF elimination in areas with logistical challenges and limited resources. Additionally, the study emphasized the importance of sampling adults (rather than young children) post-MDA, indicated that CFA is not suitable for monitoring the reduction in LF transmission with MDA with IDA, and highlighted the value of geostatistical modeling in identifying high-risk locations for LF to optimize monitoring and evaluation efforts for LF elimination programs.

## Supporting information

S1 Table Total CFA and MF prevalence across 49 villages pre-MDA. (DOCX)

S2 Table **Table A**. Summary of LF infection parameters in children and adults pre-MDA. **Table B**. Total CFA and MF prevalence in 6- 9 year age-group pre-MDA. **Table C**. Total CFA and MF prevalence in ≥10 year age-group at pre-MDA. **Table D**. Total CFA and MF prevalence in 10-17 year age-group at pre-MDA. **Table E**. Total CFA and MF prevalence in ≥ 18 years at pre-MDA.
(DOCX)

S3 Table Infection parameters in children and adults in PPS selected villages. (DOCX)

S4 Table Infection parameters in children and adults in purposively selected villages pre-MDA. (DOCX)

S5 Table Administrative coverage of IDA in ENBP November 2019. (DOCX)

S6 Table Total CFA and MF prevalence across 47 villages at 1 year post-MDA. (DOCX)

S7 Table Table A. Summary of LF Infection parameters of children and adults 1 year post-MDA. **Table B**. Total CFA and MF prevalence in 6- 9 year age-group post-MDA for each village. **Table C**. Total CFA and MF prevalence in >18 year age-group post-MDA for each village.
(DOCX)

S1 Fig Results of a coverage survey of 45 clusters and 450 households representing 2,598 children and adults. The numbers above the bars represent the sample size for each stratum and sex. The dashed line indicates the 80% recommended coverage goal for a highly efficient MDA.
(DOCX)

## References

[1] Nutman TB. Insights into the pathogenesis of disease in human lymphatic filariasis. Lymphat Res Biol. 2013;11(3):144–8. doi: 10.1089/lrb.2013.0021 24044755 PMC3780283

[2] WHO. Progress Report 2000-2009 and Strategic Plan 2010-2020. WHO; 2010.

[3] WHO. Lymphatic filariasis: Key facts 2020. Available from: https://www.who.int/news-room/fact-sheets/detail/lymphaticfilariasis#:~:text=893%20million%20people%20in%2049,and%20incapacitated%20by%20the%20disease.

[4] NTD Modelling Consortium Lymphatic Filariasis Group. The roadmap towards elimination of lymphatic filariasis by 2030: insights from quantitative and mathematical modelling. Gates Open Res. 2019;3:1538. doi: 10.12688/gatesopenres.13065.1 31728440 PMC6833911

[5] Lymphatic filariasis - managing morbidity and preventing disability: an aide-mémoire for national programme managers. 2nd edition. Geneva: World Health Organization; 2021.

[6] Stolk WA, Swaminathan S, van Oortmarssen GJ, Das PK, Habbema JDF. Prospects for elimination of bancroftian filariasis by mass drug treatment in Pondicherry, India: a simulation study. J Infect Dis. 2003;188(9):1371–81. doi: 10.1086/378354 14593597

[7] World Health Organization. The Global Programme to Eliminate Lymphatic Filariasis: Progress Report 2000-2009 and Strategic Plan 2010-2020. World Health Organization. 2011.

[8] WHO. Global programme to eliminate lymphatic filariasis: progress report, 2023. Geneva: World Health Organization. 2024.

[9] World Health Organization. Guideline: Alternative mass drug administration regimens to eliminate lymphatic filariasis. World Health Organization. 2017. 29565523

[10] King CL, Suamani J, Sanuku N, Cheng Y-C, Satofan S, Mancuso B, et al. A trial of a triple-drug treatment for lymphatic filariasis. N Engl J Med. 2018;379(19):1801–10. doi: 10.1056/NEJMoa1706854 30403937 PMC6194477

[11] King CL, Weil GJ, Kazura JW. Single-dose triple-drug therapy for Wuchereria bancrofti - 5-year follow-up. N Engl J Med. 2020;382(20):1956–7. doi: 10.1056/NEJMc1914262 32402169 PMC7175637

[12] Thomsen EK, Sanuku N, Baea M, Satofan S, Maki E, Lombore B, et al. Efficacy, safety, and pharmacokinetics of coadministered Diethylcarbamazine, Albendazole, and Ivermectin for treatment of Bancroftian Filariasis. Clin Infect Dis. 2016;62(3):334–41. doi: 10.1093/cid/civ882 26486704

[13] Laman M, Tavul L, Karl S, Kotty B, Kerry Z, Kumai S, et al. Mass drug administration of ivermectin, diethylcarbamazine, plus albendazole compared with diethylcarbamazine plus albendazole for reduction of lymphatic filariasis endemicity in Papua New Guinea: a cluster-randomised trial. Lancet Infect Dis. 2022;22(8):1200–9. doi: 10.1016/S1473-3099(22)00026-3 35533701 PMC9300473

[14] Jambulingam P, Kuttiatt VS, Krishnamoorthy K, Subramanian S, Srividya A, Raju HKK, et al. An open label, block randomized, community study of the safety and efficacy of co-administered ivermectin, diethylcarbamazine plus albendazole vs. diethylcarbamazine plus albendazole for lymphatic filariasis in India. PLoS Negl Trop Dis. 2021;15(2):e0009069. doi: 10.1371/journal.pntd.0009069 33591979 PMC7909694

[15] Bjerum CM, Ouattara AF, Aboulaye M, Kouadio O, Marius VK, Andersen BJ, et al. Efficacy and safety of a single dose of Ivermectin, Diethylcarbamazine, and Albendazole for treatment of lymphatic filariasis in Côte d’Ivoire: an open-label randomized controlled trial. Clin Infect Dis. 2020;71(7):e68–75. doi: 10.1093/cid/ciz1050 31641754 PMC7583415

[16] Guideline: Alternative Mass Drug Administration Regimens to Eliminate Lymphatic Filariasis. Geneva: World Health Organization; 2017. Report No.: Licence: CC BY-NC-SA 3.0 IGO. 29565523

[17] Transmission assessment surveys in the Global Programme to Eliminate Lymphatic Filariasis: WHO position statement. Wkly Epidemiol Rec. 2012;87(48):478–82. 23213667

[18] Organization WH. Monitoring Drug Coverage for Preventive Chemotherapy. Geneva: WHO; 2010.

[19] Preventive chemotherapy: tools for improving the quality of reported data and information: a field manual for implementation. Geneva: WHO; 2019.

[20] Arica SG, Arica V, Onur H, Gülbayzar S, Dağ H, Obut Ö. Knowledge, attitude and response of mothers about fever in their children. Emerg Med J. 2012;29(12):e4. doi: 10.1136/emermed-2011-200352 22158536

[21] Green LW. Manual for scoring socioeconomic status for research on health behavior. Public Health Rep (1896). 1970;85(9):815–27. doi: 10.2307/4593972 4989476 PMC2031767

[22] Weil GJ, Kastens W, Susapu M, Laney SJ, Williams SA, King CL, et al. The impact of repeated rounds of mass drug administration with diethylcarbamazine plus albendazole on bancroftian filariasis in Papua New Guinea. PLoS Negl Trop Dis. 2008;2(12):e344. doi: 10.1371/journal.pntd.0000344 19065257 PMC2586652

[23] Chipeta MG, Terlouw DJ, Phiri KS, Diggle PJ. Adaptive geostatistical design and analysis for prevalence surveys. Spat Stat. 2016;15:70–84. doi: 10.1016/j.spasta.2015.12.004

[24] Giorgi E, Diggle P. PrevMap: an R package for prevalence mapping. J Stat Softw. 2017;78(8):29.

[25] R Core Team. R: A Language and Environment for Statistical Computing. Vienna: R Foundation for Statistical Computing. 2023. https://www.R-project.org

[26] Graul C. LeafletR: interactive web-maps based on the leaflet javascript library. 2016.

[27] DIVA-GIS, inventorFree Spatial Data by Country. Available from: http://www.diva-gis.org/gdata2017

[28] Runfola D, Anderson A, Baier H, Crittenden M, Dowker E, Fuhrig S, et al. geoBoundaries: a global database of political administrative boundaries. PLoS One. 2020;15(4):e0231866. doi: 10.1371/journal.pone.0231866 32330167 PMC7182183

[29] Diggle PJ, Giorgi E. Model-based geostatistics for global public health: methods and application. In: Keidling N, Morgan B, Wikle C, van der Heijden P, editors. CRC Press; 2019.

[30] Eneanya OA, Fronterre C, Anagbogu I, Okoronkwo C, Garske T, Cano J, et al. Mapping the baseline prevalence of lymphatic filariasis across Nigeria. Parasit Vectors. 2019;12(1):440. doi: 10.1186/s13071-019-3682-6 31522689 PMC6745770

[31] Mwase ET, Stensgaard A-S, Nsakashalo-Senkwe M, Mubila L, Mwansa J, Songolo P, et al. Mapping the geographical distribution of lymphatic filariasis in Zambia. PLoS Negl Trop Dis. 2014;8(2):e2714. doi: 10.1371/journal.pntd.0002714 24587466 PMC3930513

[32] Slater H, Michael E. Mapping, bayesian geostatistical analysis and spatial prediction of lymphatic filariasis prevalence in Africa. PLoS One. 2013;8(8):e71574. doi: 10.1371/journal.pone.0071574 23951194 PMC3741112

[33] Diggle PJ, Amoah B, Fronterre C, Giorgi E, Johnson O. Rethinking neglected tropical disease prevalence survey design and analysis: a geospatial paradigm. Trans R Soc Trop Med Hyg. 2021;115(3):208–10. doi: 10.1093/trstmh/trab020 33587142 PMC7946792

