# Supplementary material for: Alternative approaches for monitoring and evaluation of lymphatic filariasis following mass drug treatment with ivermectin, diethylcarbamazine and albendazole in East New Britain Province, Papua New Guinea
Source: PLoS Negl Trop Dis. 2025 Jan 27;19(1):e0012128. doi: 10.1371/journal.pntd.0012128 (PMC11798438; doi:10.1371/journal.pntd.0012128)
Supplement: S1 Table — (DOCX) [file pntd.0012128.s001.docx]

**S1 Table. Total CFA and MF prevalence across 49 villages pre-MDA.**

| **District** | **Village** | **N** | **Female (N)** | **Female %** | **CFA (N)** | **CFA % (95% CI)** | **MF (N)** | **MF % (95% CI)** |
| --- | --- | --- | --- | --- | --- | --- | --- | --- |
| Kokopo | **Balanataman** | **74** | **41** | **55.41** | **0** | **-** | **0** |  |
|  | Ganai | 83 | 50 | 60.24 | 4 | 4.82 (1.3-11.9) | 0 |  |
|  | **Kababiai (Doy)** | **97** | **51** | **52.58** | **2** | **2.06 (0.3-7.3)** | **1** | **1.03 (0.03-5.6)** |
|  | **Kabilomo (Doy)** | **83** | **51** | **61.45** | **1** | **1.2 (0.03-6.5)** | **0** |  |
|  | *Karawara (Doy)* | *82* | *49* | *59.76* | *22* | *26.83 (17.6-37.8)* | *6* | *7.32 (2.7-15.3)* |
|  | **Malakuna** | **100** | **49** | **49** | **0** | **-** | **0** |  |
|  | **Palavirua** | **79** | **42** | **53.16** | **1** | **1.27 (0.03-6.9)** | **0** |  |
|  | **Ralubang** | **79** | **46** | **58.97** | **0** | **-** | **0** |  |
|  | *Utuwan (Doy)* | *101* | *66* | *65.35* | *37* | *36.63 (27.3-46.8)* | *22* | *21.78 (14.2-31.1)* |
|  | **Vunamami2** | **89** | **55** | **61.8** | **0** | **-** | **0** |  |
|  | **Vunatagia** | **90** | **43** | **47.78** | **2** | **2.22 (0.3-7.8)** | **0** |  |
| Gazelle | **Bitakapuk3** | **74** | **46** | **62.16** | **0** | **-** | **0** |  |
|  | **Kadaulung** | **96** | **47** | **48.96** | **0** | **-** | **0** |  |
|  | Karo | 99 | 61 | 61.62 | 10 | 10.1 (4.9-17.8) | 0 |  |
|  | **Kikitabu** | **86** | **43** | **50** | **0** | **-** | **0** |  |
|  | *Lan* | *101* | *63* | *62.38* | *3* | *2.97 (0.6-8.4)* | *0* |  |
|  | Matanakunai | 86 | 49 | 56.98 | 3 | 3.49 (0.7-9.9) | 0 |  |
|  | Mobilim | 82 | 41 | 50 | 3 | 3.66 (0.8-10.3) | 0 |  |
|  | **Napapar1** | **91** | **50** | **54.95** | **5** | **5.49 (1.8-12.4)** | **3** | **3.3 (0.7-9.3)** |
|  | Navui | 32 | 13 | 40.63 | 0 | - | 0 |  |
|  | **Ratavul** | **97** | **48** | **49.48** | **0** | **-** | **0** |  |
|  | **Takekel** | **102** | **55** | **53.92** | **1** | **0.98 (0.02-5.3)** | **0** |  |
|  | **Tavilo** | **85** | **43** | **50.59** | **0** | **-** | **0** |  |
|  | **Vunairoto** | **72** | **41** | **56.94** | **0** | **-** | **0** |  |
|  | **Vunapalinding1** | **83** | **49** | **59.04** | **5** | **6.02 (1.9-13.5)** | **2** | **2.41 (0.3-8.4)** |
|  | Warakindam | 101 | 60 | 59.41 | 8 | 7.92 (3.5-15.0) | 1 | 0.99 (0.03-5.4) |
|  | **Watwat** | **105** | **56** | **53.33** | **2** | **1.9 (0.2-6.7)** | **0** |  |
|  | **Yayem** | **95** | **47** | **49.47** | **0** | **-** | **0** |  |
| Pomio | Dadul | 89 | 50 | 56.18 | 0 | - | 0 |  |
|  | Gar | 94 | 52 | 55.32 | 2 | 2.13 (0.3-7.5) | 0 |  |
|  | **Hoiya** | **85** | **46** | **54.12** | **23** | **27.06 (17.9-37.8)** | **3** | **3.53 (0.7-9.9)** |
|  | Illi | 59 | 30 | 50.85 | 3 | 5.08 (1.1-14.2) | 0 |  |
|  | **Katap** | **64** | **33** | **52.38** | **0** | **-** | **0** |  |
|  | Kaukum | 88 | 48 | 54.55 | 16 | 18.18 (10.8-27.8) | 2 | 2.27 (0.3-7.9) |
|  | Kavudemki | 60 | 32 | 53.33 | 10 | 16.67 (8.3-28.5) | 1 | 1.67 (0.04-8.9) |
|  | *Lamarian* | *94* | *51* | *54.26* | *5* | *5.32 (1.8-11.9)* | *2* | *2.13 (0.3-7.5)* |
|  | *Lat* | *114* | *65* | *57.02* | *16* | *14.04 (8.2-21.8)* | *1* | *0.88 (0.02-4.8)* |
|  | **Marunga** | **94** | **51** | **54.26** | **1** | **1.06 (0.03-5.8)** | **0** |  |
|  | **Masarau** | **89** | **41** | **46.07** | **7** | **7.87 (3.2-15.5)** | **3** | **3.37 (0.7-9.5)** |
|  | Mazo | 119 | 70 | 58.82 | 2 | 1.68 (0.2-5.9) | 0 |  |
|  | Pulpul | 101 | 55 | 54.46 | 3 | 2.97 (0.62-8.4) | 0 |  |
|  | Riete | 103 | 54 | 52.43 | 2 | 1.94 (0.24-6.8) | 2 | 1.94 (0.2-6.8) |
|  | **Sivauna** | **66** | **33** | **50** | **2** | **3.03 (0.4-10.5)** | **1** | **1.52 (0.04-8.2)** |
|  | Tokai | 89 | 47 | 52.81 | 8 | 8.99 (3.9-16.9) | 1 | 1.12 (0.03-6.1) |
| Rabaul | **Livuan** | **102** | **55** | **54.46** | **1** | **0.98 (0.02-5.3)** | **0** |  |
|  | **Malaguna3** | **94** | **61** | **64.89** | **1** | **1.06 (0.03-5.8)** | **0** |  |
|  | Matupit | 68 | 44 | 64.71 | 0 | - | 0 |  |
|  | Tavui1 | 64 | 34 | 53.13 | 0 | - | 0 |  |
|  | **Volavolo** | **72** | **39** | **54.17** | **2** | **2.78 (0.34-9.7)** | **1** | **1.39 (0.04-7.5)** |

Bold lettered villages are randomly selected by population proportionate sampling (PPS)

Italicized villages are 5 purposively selected by the East New Britain LF task.
