# Supplementary material for: Alternative approaches for monitoring and evaluation of lymphatic filariasis following mass drug treatment with ivermectin, diethylcarbamazine and albendazole in East New Britain Province, Papua New Guinea
Source: PLoS Negl Trop Dis. 2025 Jan 27;19(1):e0012128. doi: 10.1371/journal.pntd.0012128 (PMC11798438; doi:10.1371/journal.pntd.0012128)
Supplement: S2 Table — Table A. Summary of LF infection parameters in children and adults pre-MDA. Table B. Total CFA and MF prevalence in 6- 9 year age-group pre-MDA. Table C. Total CFA and MF prevalence in ≥10 year age-group at pre-MDA. Table D. Total CFA and MF prevalence in 10-17 year age-group at pre-MDA. Table E. Total CFA and MF prevalence in ≥ 18 years at pre-MDA. (DOCX) [file pntd.0012128.s002.docx]

**S2 Table.**

**Table A. Summary of LF infection parameters in children and adults pre-MDA.**

|  | | **6-9 years** | | | | | **≥10 years** | | | |
| --- | --- | --- | --- | --- | --- | --- | --- | --- | --- | --- |
| **District** | **Population** | **CFA+ (N)** | **CFA %**  **(95% CI)** | **MF + (N)** | **MF %**  **(95% CI)** | **N** | **CFA +(N)** | **CFA %**  **(95% CI)** | **MF (N)** | **MF % (95% CI)** |
| Kokopo | 480 | 23 | 4.79  (3.1- 7.1) | 5 | 1.04  (0.3-2.4) | 827 | 30 | 3.6  (2.5-5.1) | 6 | 0.7  (0.3-1.6) |
| Gazelle | 660 | 10 | 1.52  (0.7-2.8) | 0 | - | 477 | 46 | 9.6  (7.2-12.7) | 24 | 5.0  (3.3-7.4) |
| Pomio | 581 | 4 | 0.69  (0.2-1.8) | 0 | - | 827 | 96 | 11.6  (9.5-13.9) | 16 | 1.9  (1.1-3.1) |
| Rabaul | 185 | 0 | - | 0 | - | 215 | 4 | 1.9  (0.5-4.7) | 1 | 0.5  (0.0-2.6) |
| **Total** | **1906** | **37** | **1.9**  **(1.4-2.7)** | **5** | **0.3**  **(0.1-0.6)** | **2346** | **176** | **7.5**  **(6.5-8.6)** | **47** | **2.0**  **(1.5-2.7)** |

**Table B. Total CFA and MF prevalence in 6- 9 year age-group pre-MDA.**

| District | Villages | N | F | %F | CFA (N) | CFA % 95% CI | MF (N) | MF % |
| --- | --- | --- | --- | --- | --- | --- | --- | --- |
| Kokopo | Balanataman | 48 | 23 | 47.92 | 0 |  |  |  |
|  | Ganai | 38 | 15 | 39.47 | 0 |  |  |  |
|  | Kababiai | 46 | 22 | 47.83 | 0 |  |  |  |
|  | Kabilomo | 35 | 19 | 54.29 | 0 |  |  |  |
|  | Karawara | 51 | 24 | 47.06 | 11 | 21.57 (11.3-35.3) | 1 | 1.96 (0.1-10.5) |
|  | Malakuna | 48 | 18 | 37.50 | 0 |  |  |  |
|  | Palavirua | 36 | 14 | 38.89 | 1 | 2.78 (0.1-14.5) |  |  |
|  | Ralubang | 35 | 17 | 48.57 | 0 |  |  |  |
|  | Utuwan | 51 | 29 | 56.86 | 11 | 21.57 (11.3-35.3) | 4 | 7.84 (2.2-18.9) |
|  | Vunamami2 | 61 | 36 | 59.02 | 0 |  |  |  |
|  | Vunatagia | 31 | 10 | 32.26 | 0 |  |  |  |
| Gazelle | Bitakapuk3 | 29 | 19 | 65.52 | 0 |  |  |  |
|  | Kadaulung | 16 | 8 | 50.00 | 0 |  |  |  |
|  | Karo | 48 | 27 | 56.25 | 2 | 4.17 (0.5-14.3) |  |  |
|  | Kikitabu | 36 | 12 | 33.33 | 0 |  |  |  |
|  | Lan | 50 | 28 | 56.00 | 1 | 2 (0.1-10.7) |  |  |
|  | Matanakunai | 31 | 15 | 48.39 | 0 |  |  |  |
|  | Mobilim | 26 | 15 | 57.69 | 0 |  |  |  |
|  | Napapar1 | 41 | 21 | 51.22 | 1 | 2.44 (0.1-12.9) |  |  |
|  | Navui | 32 | 13 | 40.63 | 0 |  |  |  |
|  | Ratavul | 49 | 22 | 44.90 | 0 |  |  |  |
|  | Takekel | 52 | 29 | 55.77 | 1 | 1.92 (0.1-10.3) |  |  |
|  | Tavilo | 27 | 13 | 48.15 | 0 |  |  |  |
|  | Vunairoto | 34 | 17 | 50.00 | 0 |  |  |  |
|  | Vunapalinding1 | 50 | 26 | 52.00 | 3 | 6 (1.3-16.6) |  |  |
|  | Warakindam | 53 | 28 | 52.83 | 2 | 3.77 (0.5-12.9) |  |  |
|  | Watwat | 51 | 23 | 45.10 | 0 |  |  |  |
|  | Yayem | 35 | 19 | 54.29 | 0 |  |  |  |
| Pomio | Dadul | 23 | 7 | 30.43 | 0 |  |  |  |
|  | Gar | 43 | 20 | 46.51 | 1 | 2.33 (0.1-12.3) |  |  |
|  | Hoiya | 28 | 14 | 50.00 | 2 | 7.14 (0.88-23.5) |  |  |
|  | Illi | 19 | 8 | 42.11 | 0 |  |  |  |
|  | Katap | 17 | 9 | 52.94 | 0 |  |  |  |
|  | Kaukum | 39 | 18 | 46.15 | 1 | 2.56 (0.1-13.5) |  |  |
|  | Kavudemki | 26 | 9 | 34.62 | 0 |  |  |  |
|  | Lamarian | 42 | 17 | 40.48 | 0 |  |  |  |
|  | Lat | 34 | 17 | 50.00 | 0 |  |  |  |
|  | Marunga | 34 | 16 | 47.06 | 0 |  |  |  |
|  | Masarau | 37 | 16 | 43.24 | 0 |  |  |  |
|  | Mazo | 66 | 30 | 45.45 | 0 |  |  |  |
|  | Pulpul | 50 | 21 | 42.00 | 0 |  |  |  |
|  | Riete | 43 | 16 | 37.21 | 0 |  |  |  |
|  | Sivauna | 29 | 15 | 51.72 | 0 |  |  |  |
|  | Tokai | 51 | 27 | 52.94 | 0 |  |  |  |
| Rabaul | Livuan | 50 | 25 | 50.00 | 0 |  |  |  |
|  | Malaguna3 | 46 | 26 | 56.52 | 0 |  |  |  |
|  | Matupit | 30 | 19 | 63.33 | 0 |  |  |  |
|  | Tavui1 | 28 | 13 | 46.43 | 0 |  |  |  |
|  | Volavolo | 31 | 13 | 41.94 | 0 |  |  |  |

**Table C. Total CFA and MF prevalence in ≥10 year age-group at pre-MDA.**

| **District** | **Village** | **N** | **F** | **%F** | **CFA (N)** | **CFA % (95% CI)** | **MF (N)** | **MF % (95% CI)** |
| --- | --- | --- | --- | --- | --- | --- | --- | --- |
| Kokopo | Balanataman | 26 | 18 | 69.23 | 0 |  |  |  |
|  | Ganai | 45 | 35 | 77.78 | 4 | 8.89 (2.5-21.2) | 0 |  |
|  | Kababiai | 51 | 29 | 56.86 | 2 | 3.92 (0.5-13.5) | 1 | 1.96 (0.1-10.5) |
|  | Kabilomo | 48 | 32 | 66.67 | 1 | 2.08 (0.1-11.1) |  |  |
|  | Karawara | 31 | 25 | 80.65 | 11 | 35.48 (19.2-54.6) | 5 | 16.13 (5.5-33.7) |
|  | Malakuna | 52 | 31 | 59.62 | 0 |  |  |  |
|  | Palavirua | 43 | 28 | 65.12 | 0 |  |  |  |
|  | Ralubang | 44 | 29 | 65.91 | 0 |  |  |  |
|  | Utuwan | 50 | 37 | 74.00 | 26 | 52 (37.4-66.3) | 18 | 36 (22.9-50.8) |
|  | Vunamami2 | 28 | 19 | 67.86 |  |  |  |  |
|  | Vunatagia | 59 | 33 | 55.93 | 2 | 3.39 (2-3.4) | 0 |  |
| Gazelle  ‘ | Bitakapuk3 | 45 | 27 | 60.00 |  |  |  |  |
|  | Kadaulung | 80 | 39 | 48.75 |  |  |  |  |
|  | Karo | 51 | 34 | 66.67 | 8 | 15.69 (7.0-28.6) |  |  |
|  | Kikitabu | 50 | 31 | 62.00 |  |  |  |  |
|  | Lan | 51 | 35 | 68.63 | 2 | 3.92 (0.5- 13.5) | 0 |  |
|  | Matanakunai | 55 | 34 | 61.82 | 3 | 5.45 (1.1-15.1) | 0 |  |
|  | Mobilim | 56 | 26 | 46.43 | 3 | 5.36 (1.1-14.9) | 0 |  |
|  | Napapar1 | 50 | 29 | 58.00 | 4 | 8 (2.2-19.2) | 3 | 6 (1.3-16.6) |
|  | Navui | 0 | 0 | - |  |  |  |  |
|  | Ratavul | 48 | 26 | 54.17 |  |  |  |  |
|  | Takekel | 50 | 26 | 52.00 |  |  |  |  |
|  | Tavilo | 58 | 30 | 51.72 |  |  |  |  |
|  | Vunairoto | 38 | 24 | 63.16 |  |  |  |  |
|  | Vunapalinding1 | 33 | 23 | 69.70 | 2 | 6.06 (2-6.1) | 2 | 6.06 (0.7-20.2) |
|  | Warakindam | 48 | 32 | 66.67 | 6 | 12.5 (4.7-25.3) | 1 | 2.08 (0.1-11.1) |
|  | Watwat | 54 | 33 | 61.11 | 2 | 3.7 (0.5-12.8) | 0 |  |
|  | Yayem | 60 | 28 | 46.67 |  |  |  |  |
| Pomio | Dadul | 66 | 43 | 65.15 |  |  |  |  |
|  | Gar | 51 | 43 | 84.31 | 1 | 1.96 (0.1-10.5) | 0 |  |
|  | Hoiya | 57 | 32 | 56.14 | 21 | 36.84 (24.5-50.7) | 3 | 5.26 (1.1-14.6) |
|  | Illi | 40 | 22 | 55.00 | 3 | 7.5 (1.6-20.4) | 0 |  |
|  | Katap | 47 | 24 | 51.06 |  |  |  |  |
|  | Kaukum | 49 | 30 | 61.22 | 15 | 30.61 (18.3-45.4) | 2 | 4.08 (0.5-13.9) |
|  | Kavudemki | 34 | 23 | 67.65 | 10 | 29.41 (15.1-47.5) | 1 | 2.94 (0.1-15.3) |
|  | Lamarian | 52 | 34 | 65.38 | 5 | 9.62 (3.2-21.0) | 2 | 3.85 (0.5-13.2) |
|  | Lat | 80 | 48 | 60.00 | 16 | 20 (11.9-30.4) | 1 | 1.25 (0.0-6.8) |
|  | Marunga | 60 | 35 | 58.33 | 1 | 1.67 (0.0-8.9) | 0 |  |
|  | Masarau | 52 | 25 | 48.08 | 7 | 13.46 (5.6-25.8) | 3 | 5.77 (1.2-15.9) |
|  | Mazo | 53 | 40 | 75.47 | 2 | 3.77 (0.5-12.9) | 0 |  |
|  | Pulpul | 51 | 34 | 66.67 | 3 | 5.88 (1.2-16.2) | 0 |  |
|  | Riete | 60 | 38 | 63.33 | 2 | 3.33 (0.4-11.5) | 2 | 3.33 (0.4-11.5) |
|  | Sivauna | 37 | 18 | 48.65 | 2 | 5.41 (0.7-18.2) | 1 | 2.7 (0.1-14.2) |
|  | Tokai | 38 | 20 | 52.63 | 8 | 21.05 (9.6-37.3) | 1 | 2.63 (0.1-13.8) |
| Rabaul | Livuan | 52 | 30 | 57.69 | 1 | 1.92 (0.1-10.3) | 0 |  |
|  | Malaguna3 | 48 | 35 | 72.92 | 1 | 2.08 (0.1-11.1) | 0 |  |
|  | Matupit | 38 | 25 | 65.79 |  |  |  |  |
|  | Tavui1 | 36 | 21 | 58.33 |  |  |  |  |
|  | Volavolo | 41 | 26 | 63.41 | 2 | 4.88 (0.6-16.5) | 1 | 2.44 (0.1-12.9) |

**Table D. Total CFA and MF prevalence in 10-17 year age-group at pre-MDA.**

| **District** | **Village** | **N** | **F** | **%F** | **CFA (N)** | **CFA % (95% CI)** | **MF (N)** | **MF % (95% CI)** |
| --- | --- | --- | --- | --- | --- | --- | --- | --- |
| Kokopo | Balanataman | 5 | 1 | 20.00 | 0 |  |  |  |
|  | Ganai | 14 | 10 | 71.43 | 1 | 7.14 (0.2-33.9) | 0 |  |
|  | Kababiai | 15 | 6 | 40.00 | 0 |  |  |  |
|  | Kabilomo | 11 | 7 | 63.64 | 1 | 9.1 (0.2-41.3) | 0 |  |
|  | Karawara | 11 | 9 | 81.82 | 4 | 36.4 (10.9- 69.2) | 3 | 27.27 (6.0-60.9) |
|  | Malakuna | 27 | 12 | 44.44 | 0 |  |  |  |
|  | Palavirua | 15 | 13 | 86.67 | 0 |  |  |  |
|  | Ralubang | 9 | 3 | 33.33 | 0 |  |  |  |
|  | Utuwan | 12 | 7 | 58.33 | 3 | 25 (5.5-57.2) | 2 | 16.67 (2.1-48.4) |
|  | Vunamami2 | 4 | 1 | 25.00 | 0 |  |  |  |
|  | Vunatagia | 15 | 10 | 66.67 | 0 |  |  |  |
| Gazelle | Bitakapuk3 | 13 | 8 | 61.54 | 0 |  |  |  |
|  | Kadaulung | 14 | 8 | 57.14 | 0 |  |  |  |
|  | Karo | 8 | 3 | 37.50 | 0 |  |  |  |
|  | Kikitabu | 13 | 7 | 53.85 | 0 |  |  |  |
|  | Lan | 5 | 3 | 60.00 | 0 |  |  |  |
|  | Matanakunai | 7 | 2 | 28.57 | 0 |  |  |  |
|  | Mobilim | 18 | 9 | 50.00 | 1 | 5.56 (0.1-27.3) | 0 |  |
|  | Napapar1 | 15 | 5 | 33.33 | 1 | 6.67 (0.2-31.9) | 0 |  |
|  | Navui | 0 | 0 | - | - |  |  |  |
|  | Ratavul | 11 | 6 | 54.55 | 0 |  |  |  |
|  | Takekel | 16 | 8 | 50.00 | 0 |  |  |  |
|  | Tavilo | 15 | 5 | 33.33 | 0 |  |  |  |
|  | Vunairoto | 11 | 4 | 36.36 | 0 |  |  |  |
|  | Vunapalinding1 | 5 | 2 | 40.00 | 0 |  |  |  |
|  | Warakindam | 15 | 11 | 73.33 | 0 |  |  |  |
|  | Watwat | 15 | 7 | 46.67 | 1 | 6.67 (0.2-31.9) | 0 |  |
|  | Yayem | 23 | 11 | 47.83 | 0 |  |  |  |
| Pomio | Dadul | 18 | 11 | 61.11 | 0 |  |  |  |
|  | Gar | 16 | 8 | 50.00 | 0 |  |  |  |
|  | Hoiya | 19 | 9 | 47.37 | 4 | 21.05 (6.1- 45.6) | 1 | 5.26 (0.1-26.0) |
|  | Illi | 15 | 9 | 60.00 | 1 | 6.67 (0.2- 31.9) |  |  |
|  | Katap | 25 | 13 | 52.00 | 0 |  |  |  |
|  | Kaukum | 16 | 11 | 68.75 | 3 | 18.75 (4.1-45.7) | 0 |  |
|  | Kavudemki | 10 | 4 | 40.00 | 2 | 20 (2.5-55.6) | 0 |  |
|  | Lamarian | 11 | 5 | 45.45 | 0 |  |  |  |
|  | Lat | 28 | 17 | 60.71 | 0 |  |  |  |
|  | Marunga | 16 | 10 | 62.50 | 0 |  |  |  |
|  | Masarau | 6 | 1 | 16.67 | 0 |  |  |  |
|  | Mazo | 1 | 1 | 100.00 | 0 |  |  |  |
|  | Pulpul | 19 | 12 | 63.16 | 0 |  |  |  |
|  | Riete | 14 | 9 | 64.29 | 0 |  |  |  |
|  | Sivauna | 11 | 4 | 36.36 | 0 |  |  |  |
|  | Tokai | 11 | 5 | 45.45 | 0 |  |  |  |
| Rabaul | Livuan | 24 | 13 | 54.17 | 0 |  |  |  |
|  | Malaguna3 | 5 | 2 | 40.00 | 0 |  |  |  |
|  | Matupit | 17 | 10 | 58.82 | 0 |  |  |  |
|  | Tavui1 | 9 | 5 | 55.56 | 0 |  |  |  |
|  | Volavolo | 20 | 9 | 45.00 | 1 | 5 (0.1- 24.9) | 0 |  |

**Table E. Total CFA and MF prevalence in ≥18 years at pre-MDA.**

| **District** | **Village** | **N** | **F** | **%F** | **CFA (N)** | **CFA % (95% CI)** | **MF (N)** | **MF % (95% CI)** |
| --- | --- | --- | --- | --- | --- | --- | --- | --- |
| Kokopo | Balanataman | 21 | 17 | 80.95 | 0 |  |  |  |
|  | Ganai | 31 | 25 | 80.65 | 3 | 9.68 (2.0-25.8) | 0 |  |
|  | Kababiai | 36 | 23 | 63.89 | 2 | 5.56 (0.7-18.7) | 1 | 2.78 (0.1-14.5) |
|  | Kabilomo | 37 | 25 | 67.57 | 0 |  |  |  |
|  | Karawara | 20 | 16 | 80.00 | 7 | 35 (15.4-59.2) | 2 | 10 (1.2-31.7) |
|  | Malakuna | 25 | 19 | 76.00 | 0 |  |  |  |
|  | Palavirua | 28 | 15 | 53.57 | 0 |  |  |  |
|  | Ralubang | 35 | 26 | 74.29 | 0 |  |  |  |
|  | Utuwan | 38 | 30 | 78.95 | 23 | 60.53 (43.4-75.9) | 16 | 42.11 (26.3-59.2) |
|  | Vunamami2 | 24 | 18 | 75.00 | 0 |  |  |  |
|  | Vunatagia | 44 | 23 | 52.27 | 2 | 4.55 (0.6-15.5) |  |  |
| Gazelle | Bitakapuk3 | 32 | 19 | 59.38 | 0 |  |  |  |
|  | Kadaulung | 66 | 31 | 46.97 | 0 |  |  |  |
|  | Karo | 43 | 31 | 72.09 | 8 | 18.6 (8.4-33.4) | 0 |  |
|  | Kikitabu | 37 | 24 | 64.86 | 0 |  |  |  |
|  | Lan | 46 | 32 | 69.57 | 2 | 4.35 (0.5-14.8) | 0 |  |
|  | Matanakunai | 48 | 32 | 66.67 | 3 | 6.25 (1.3-17.2) |  |  |
|  | Mobilim | 38 | 17 | 44.74 | 2 | 5.26 (0.6-17.8) | 0 |  |
|  | Napapar1 | 35 | 24 | 68.57 | 3 | 8.57 (1.8-3.1) | 3 | 8.57 (1.8-23.1) |
|  | Navui | 0 | 0 | 0.00 | 0 |  |  |  |
|  | Ratavul | 37 | 20 | 54.05 | 0 |  |  |  |
|  | Takekel | 34 | 18 | 52.94 | 0 |  |  |  |
|  | Tavilo | 43 | 25 | 58.14 | 0 |  |  |  |
|  | Vunairoto | 27 | 20 | 74.07 | 0 |  |  |  |
|  | Vunapalinding1 | 28 | 21 | 75.00 | 2 | 7.14 (0.9-23.5) | 2 | 7.14 (0.9-23.5) |
|  | Warakindam | 33 | 21 | 63.64 | 6 | 18.18 (6.9-53.5) | 1 | 3.03 (0.1-15.8) |
|  | Watwat | 39 | 26 | 66.67 | 1 | 2.56 (0.1-13.5) |  |  |
|  | Yayem | 37 | 17 | 45.95 | 0 |  |  |  |
| Pomio | Dadul | 48 | 32 | 66.67 | 0 |  |  |  |
|  | Gar | 35 | 24 | 68.57 | 1 | 2.86 (0.1-14.9) | 0 |  |
|  | Hoiya | 38 | 23 | 60.53 | 17 | 44.74 (28.6-61.7) | 2 | 5.26 (0.6-17.8) |
|  | Illi | 25 | 13 | 52.00 | 2 | 8 (0.9-26.0) | 0 |  |
|  | Katap | 22 | 11 | 50.00 | 0 |  |  |  |
|  | Kaukum | 33 | 19 | 57.58 | 12 | 36.36 (20.4-54.9) | 2 | 6.06 (0.7-20.2) |
|  | Kavudemki | 24 | 19 | 79.17 | 8 | 33.33 (15.6-55.3) | 1 | 4.17 (0.1-21.1) |
|  | Lamarian | 41 | 29 | 70.73 | 5 | 12.2 (4.1-26.2) | 2 | 4.88 (0.6-16.5) |
|  | Lat | 52 | 31 | 59.62 | 16 | 30.77 (18.7-45.1) | 1 | 1.92 (0.1-10.3) |
|  | Marunga | 44 | 25 | 56.82 | 1 | 2.27 (0.1-12.0) | 0 |  |
|  | Masarau | 46 | 24 | 52.17 | 7 | 15.22 (6.3-28.9) | 3 | 6.52 (1.4-17.9) |
|  | Mazo | 52 | 39 | 75.00 | 2 | 3.85 (0.5-13.2) | 0 |  |
|  | Pulpul | 32 | 22 | 68.75 | 3 | 9.38 (1.9-25.0) | 0 |  |
|  | Riete | 46 | 29 | 63.04 | 2 | 4.35 (0.5-14.8) | 2 | 4.35 (0.5-14.8) |
|  | Sivauna | 26 | 14 | 53.85 | 2 | 7.69 (0.9-25.1) | 1 | 3.85 (0.1-19.6) |
|  | Tokai | 27 | 15 | 55.56 | 8 | 29.63 (13.8-50.2) | 1 | 3.7 (0.1-18.9) |
| Rabaul | Livuan | 28 | 17 | 60.71 | 1 | 3.57 (0.1-18.4) | 0 |  |
|  | Malaguna3 | 43 | 33 | 76.74 | 1 | 2.33 (0.1-12.3) | 0 |  |
|  | Matupit | 21 | 15 | 71.43 | 0 |  |  |  |
|  | Tavui1 | 27 | 16 | 59.26 | 0 |  |  |  |
|  | Volavolo | 21 | 17 | 80.95 | 1 | 4.76 (0.12-23.8) | 1 | 4.76 (0.1-23.8) |
