# Supplementary material for: Alternative approaches for monitoring and evaluation of lymphatic filariasis following mass drug treatment with ivermectin, diethylcarbamazine and albendazole in East New Britain Province, Papua New Guinea
Source: PLoS Negl Trop Dis. 2025 Jan 27;19(1):e0012128. doi: 10.1371/journal.pntd.0012128 (PMC11798438; doi:10.1371/journal.pntd.0012128)
Supplement: S3 Table — (DOCX) [file pntd.0012128.s003.docx]

**S3 Table. Infection parameters in children and adults in randomly selected villages.**

|  | **Total CFA and MF % in randomized selected villages by PPS** | | | | | **6-9 years** | | | | | **≥10 years** | | | | |
| --- | --- | --- | --- | --- | --- | --- | --- | --- | --- | --- | --- | --- | --- | --- | --- |
| **District** | **Population** | **CFA + (N)** | **CFA % (95% CI)** | **Mf (n)** | **Mf %**  **(95% CI** | **N** | **CFA + (N)** | **CFA %**  **(95% CI)** | **MF+ (N)** | **Mf %**  **(95% CI)** | **N** | **CFA +(N)** | **CFA %**  **(95% CI)** | **MF + (N)** | **MF %**  **(95% CI)** |
| **Kokopo** | 691 | 6 | 0.87  (0.3-1.9) | 1 | 0.1  (0.0-0.8) | 340 | 1 | 0.3  (0.0-1.6) | 0 | - | 351 | 5 | 1.4  (0.5-3.3) | 1 | 0.3  (0.0-1.6) |
| **Gazelle** | 1072 | 16 | 1.5  (0.9-2.4) | 5 | 0.5  (0.2-1.1) | 451 | 5 | 1.1  (0.4-2.6) | 0 | - | 621 | 11 | 1.8  (0.9-3.2) | 5 | 0.8  (0.3-1.9) |
| **Pomio** | 398 | 33 | 8.29  (5.8-11.5) | 7 | 1.8  (0.7-3.6) | 145 | 2 | 1.4  (0.2-4.9) | 0 | - | 253 | 31 | 12.3  (8.5-16.9) | 7 | 2.8  (1.1-5.6) |
| **Rabaul** | 400 | 4 | 1.0  (0.3-2.5) | 1 | 0.3  (0.0-1.4) | 185 | 0 | - | 0 | - | 215 | 4 | 1.9  (0.5-4.7) | 1 | 0.5  (0.0-2.6) |
| **Total** | **2561** | **59** | **2.3**  **(1.8-2.9)** | **14** | **0.6**  **(0.3-0.9)** | **1121** | **8** | **0.7**  **(0.3-1.4)** | **0** | **-** | **1440** | **51** | **3.5**  **(2.7-4.6)** | **14** | **0.9**  **(0.5-1.6)** |
