# Supplementary material for: Alternative approaches for monitoring and evaluation of lymphatic filariasis following mass drug treatment with ivermectin, diethylcarbamazine and albendazole in East New Britain Province, Papua New Guinea
Source: PLoS Negl Trop Dis. 2025 Jan 27;19(1):e0012128. doi: 10.1371/journal.pntd.0012128 (PMC11798438; doi:10.1371/journal.pntd.0012128)
Supplement: S4 Table — (DOCX) [file pntd.0012128.s004.docx]

**S4 Table. Infection parameters in children and adults in purposively selected villages pre-MDA.**

|  | **Total CFA and MF % in purposive selected villages** | | | | | **6-9 years** | | | | | **≥10 years** | | | | |
| --- | --- | --- | --- | --- | --- | --- | --- | --- | --- | --- | --- | --- | --- | --- | --- |
| **District** | **Popn.** | **CFA+ (N)** | **CFA %**  **(95% CI)** | **Mf + (N)** | **Mf %**  **(95% CI** | **N** | **CFA + (N)** | **CFA %**  **(95% CI)** | **MF + (N)** | **MF % (95% CI)** | **N** | **CFA+ (N)** | **CFA %**  **(95% CI)** | **MF +**  **(N)** | **MF %**  **(95% CI)** |
| Kokopo | 266 | 63 | 23.7  (18.7-29.3) | 28 | 10.5  (7.1-14.8) | 140 | 22 | 15.7  (10.1-22.8) | 5 | 3.6  (1.2-8.1) | 126 | 41 | 32.5  (24.5-41.5) | 23 | 18.3  (11.9-26.1) |
| Gazelle | 415 | 24 | 5.8  (3.7-8.5) | 1 | 0.24  (0.0-1.3) | 209 | 5 | 2.4  (0.8-5.5) | 0 | - | 206 | 19 | 9.2  (5.6-14.0) | 1 | 0.5  (0.0-2.7) |
| Pomio | 1010 | 67 | 6.6  (5.2-8.4) | 9 | 0.9  (0.4-1.7) | 436 | 2 | 0.5  (0.1-1.7) | 0 | - | 574 | 65 | 11.3  (8.9-14.2) | 9 | 1.6  (0.7-2.9) |
| Rabaul* | 0 | 0 | - | 0 | - | - | - | - | - |  | - |  |  | - |  |
| **Total** | **1691** | **154** | **9.1**  **(7.8-10.6)** | **38** | **2.3**  **(1.6-3.1)** | **785** | **29** | **3.7**  **(2.5-5.3)** | **5** | **0.6**  **(0.2-1.5)** | **906** | **125** | **13.8**  **(11.6-16.2)** | **33** | **3.6**  **(2.5-5.1)** |

*Villages in the Rabaul district were not purposively selected because they were categorized as a low-risk having <2% CFA and mf prevalence in previously surveyed villages.
