# Supplementary figures and images for: Alternative approaches for monitoring and evaluation of lymphatic filariasis following mass drug treatment with ivermectin, diethylcarbamazine and albendazole in East New Britain Province, Papua New Guinea
Source: PLoS Negl Trop Dis. 2025 Jan 27;19(1):e0012128. doi: 10.1371/journal.pntd.0012128 (PMC11798438; doi:10.1371/journal.pntd.0012128)

**S5 Table.** **Administrative coverage of IDA in ENBP November 2019.**


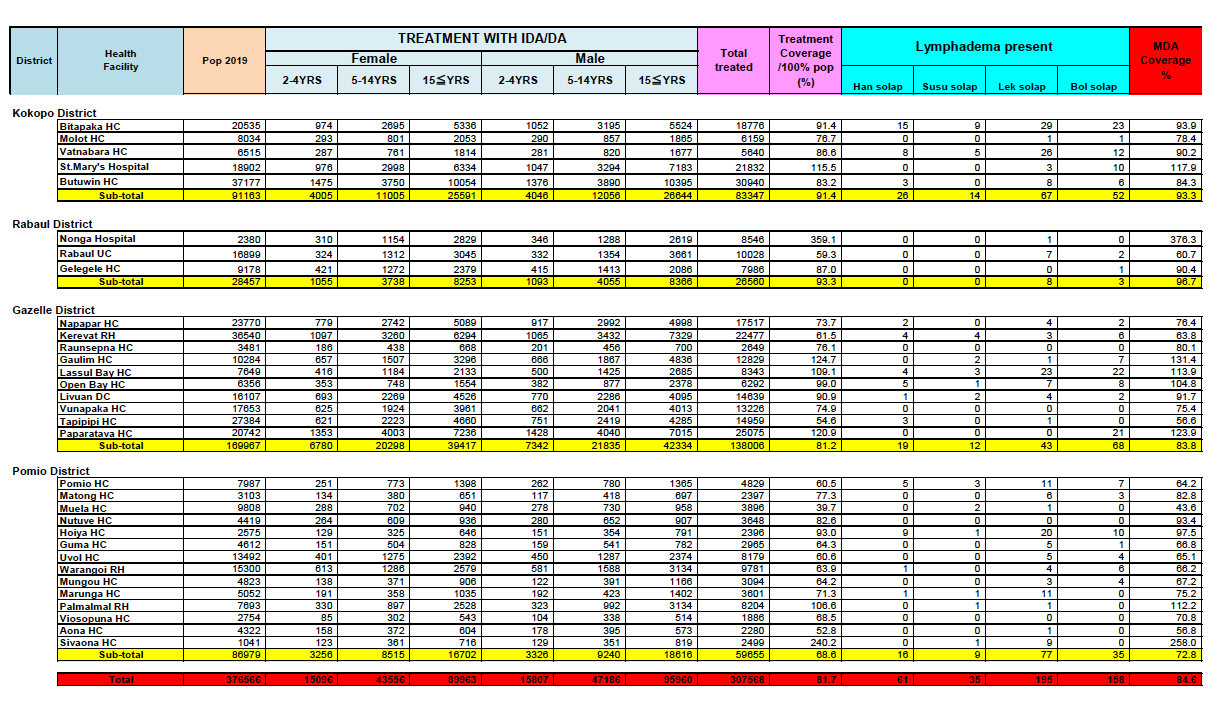

Supplement: S5 Table — (DOCX) [file pntd.0012128.s005.docx]
