# Supplementary material for: Alternative approaches for monitoring and evaluation of lymphatic filariasis following mass drug treatment with ivermectin, diethylcarbamazine and albendazole in East New Britain Province, Papua New Guinea
Source: PLoS Negl Trop Dis. 2025 Jan 27;19(1):e0012128. doi: 10.1371/journal.pntd.0012128 (PMC11798438; doi:10.1371/journal.pntd.0012128)
Supplement: S6 Table — (DOCX) [file pntd.0012128.s006.docx]

**S6 Table. Total CFA and MF prevalence across 47 villages at 1 year post-MDA.**

| **District** | **Village** | **N** | **Female (N)** | **Female %** | **CFA (N)** | **CFA % (95% CI)** | **Mf (N)** | **Mf %** |
| --- | --- | --- | --- | --- | --- | --- | --- | --- |
| Kokopo | Ganai | 100 | 69 | 69 | 16 | 16.16 (9.5-24.9) | 0 | 0 |
|  | Kababia | 100 | 57 | 57 | 6 | 6 (2.2-12.6) | 0 | 0 |
|  | *Kabatira* | 100 | 60 | 60 | 6 | 6 (2.2-12.6) | 1 | 1 (0.0-5.5) |
|  | Karawara | 100 | 47 | 47 | 22 | 22.28 (14.3-31.4) | 2 | 2 (0.2-7.0) |
|  | *Mualim* | 99 | 58 | 59.6 | 4 | 4.04 (1.11-10.02) | 0 | 0 |
|  | *Palpal* | 101 | 75 | 74.26 | 7 | 6.93 (2.8-13.8) | 0 | 0 |
|  | *Ralauna* | 58 | 37 | 63.79 | 0 | 0 | 0 | 0 |
|  | Utuwan | 100 | 57 | 57 | 36 | 36 (26.6-46.2) | 7 | 7 (2.9-13.9) |
|  | *Virian* | 100 | 63 | 63 | 3 | 3 (0.62-8.5) | 0 | 0 |
| Gazelle | *Kamanaka* | 100 | 57 | 57 | 0 | 0 | 0 | 0 |
|  | Karo | 100 | 52 | 52 | 1 | 1 (0.03-5.5) | 0 | 0 |
|  | Lan | 100 | 54 | 54 | 2 | 2 (0.24-7.04) | 0 | 0 |
|  | Matanaku | 102 | 56 | 54.9 | 3 | 2.94 (0.61-8.4) | 0 | 0 |
|  | Mobilim | 100 | 35 | 35 | 4 | 4 (1.1-9.9) | 0 | 0 |
|  | Napapar1 | 98 | 55 | 56.12 | 2 | 2.04 (0.3-7.2) | 0 | 0 |
|  | *Puktas* | 100 | 55 | 55 | 4 | 4 (1.1-9.9) | 0 | 0 |
|  | *Ragaga* | 100 | 61 | 61 | 6 | 6.06 (2.23-12.6) | 0 | 0 |
|  | *Ulak* | 99 | 52 | 52.53 | 3 | 3.03 (0.63-8.6) | 0 | 0 |
|  | Vunapala | 102 | 63 | 61.76 | 1 | 0.98 (0.02-5.3) | 0 | 0 |
|  | Warakind | 100 | 52 | 52 | 7 | 7 (2.9-13.9) | 0 | 0 |
|  | *Wuatam* | 99 | 25 | 25.25 | 2 | 2.04 (0.3-7.2) | 0 | 0 |
| Pomio | *Awatka* | 100 | 55 | 55 | 4 | 4 (1.1-9.9) | 0 | 0 |
|  | *Bogotata* | 103 | 62 | 60.19 | 13 | 12.75 (6.9-20.9) | 0 | 0 |
|  | *Buka* | 98 | 62 | 63.27 | 24 | 24.49 (16.4-34.2) | 0 | 0 |
|  | *Bulus* | 98 | 55 | 56.12 | 19 | 19.39 (12.1-28.6) | 0 | 0 |
|  | Gar | 99 | 62 | 62.63 | 0 | 0 | 0 | 0 |
|  | *Gumgum* | 100 | 45 | 45 | 6 | 6 (2.2-12.6) | 1 | 1 (0.03-5.5) |
|  | Hoiya | 99 | 55 | 55.56 | 17 | 17.17 (10.3-26.1) | 0 | 0 |
|  | Illi | 100 | 48 | 48 | 0 | 0 | 0 | 0 |
|  | *Ivai* | 98 | 74 | 75.51 | 6 | 6.12 (2.3-12.9) | 1 | 1.02 (0.0-5.6) |
|  | *Karlai* | 100 | 46 | 46 | 17 | 17 (10.2-25.8) | 0 | 0 |
|  | Kaukum | 100 | 45 | 45 | 16 | 16 (9.4-24.7) | 0 | 0 |
|  | Kavudemk | 100 | 56 | 56 | 12 | 12.12 (6.4-20.0) | 0 | 0 |
|  | *Kolai* | 93 | 51 | 54.84 | 4 | 4.3 (1.2-10.7) | 0 | 0 |
|  | Lamarian | 99 | 53 | 53.54 | 11 | 11.11 (5.6-19.01) | 0 | 0 |
|  | Lat | 160 | 74 | 46.25 | 20 | 12.5 (7.8-18.7) | 1 | 0.63 (0.0-3.4) |
|  | *Long* | 99 | 36 | 36.36 | 14 | 14.29 (7.9-22.6) | 1 | 1.01 (0.0-5.5) |
|  | Masarau | 77 | 50 | 64.94 | 5 | 6.49 (2.1-14.5) | 0 | 0 |
|  | *Milim* | 99 | 59 | 59.6 | 6 | 6.06 (2.3-12.7) | 0 | 0 |
|  | *Muu* | 99 | 48 | 48.48 | 8 | 8.08 (3.6-15.3) | 0 | 0 |
|  | *PomComSc* | 82 | 47 | 57.32 | 0 | 0 | 0 | 0 |
|  | Pulpul | 87 | 55 | 63.22 | 6 | 6.98 (2.6-14.4) | 0 | 0 |
|  | *Rainut* | 68 | 32 | 47.06 | 2 | 2.94 (0.4-10.2) | 0 | 0 |
|  | Riete | 100 | 51 | 51 | 2 | 2 (0.2-7.0) | 0 | 0 |
|  | Sivauna | 96 | 56 | 58.33 | 9 | 9.47 (4.4-17.1) | 0 | 0 |
|  | Tokai | 99 | 58 | 58.59 | 16 | 16.16 (9.5-24.9) | 0 | 0 |
| Rabaul | Tavui | 100 | 58 | 58 | 0 | 0 | 0 | 0 |
