# Supplementary material for: Alternative approaches for monitoring and evaluation of lymphatic filariasis following mass drug treatment with ivermectin, diethylcarbamazine and albendazole in East New Britain Province, Papua New Guinea
Source: PLoS Negl Trop Dis. 2025 Jan 27;19(1):e0012128. doi: 10.1371/journal.pntd.0012128 (PMC11798438; doi:10.1371/journal.pntd.0012128)
Supplement: S7 Table — Table A. Summary of LF Infection parameters of children and adults 1 year post-MDA. Table B. Total CFA and MF prevalence in 6- 9 year age-group post-MDA for each village. Table C. Total CFA and MF prevalence in >18 year age-group post-MDA for each village. (DOCX) [file pntd.0012128.s007.docx]

**S7 Table.**

**Table A. Summary of LF Infection parameters of children and adults 1 year post-MDA.**

|  | **6-9** | | | | | | **≥ 18** | | | | |
| --- | --- | --- | --- | --- | --- | --- | --- | --- | --- | --- | --- |
| **District** | **N** | **CFA (N)** | **CFA %**  **(95% CI)** | N | **MF**  **(N)** | **Mf % (95% CI)** | **N** | **CFA (N)** | **CFA %**  **(95% CI)** | **MF (N)** | **MF % (95% CI)** |
| Kokopo | 231 | 9 | 3.9  (1.8-2.3) | 231 | 0 | - | 627 | 91 | 14.5  (11.9-17.6) | 10 | 1.6  (0.8-2.9) |
| Gazelle | 93 | 1 | 1.1  (0.0-5.9) | 93 | 0 | - | 1107 | 34 | 3.1  (2.1-4.3) | 0 | 0.00 |
| Pomio | 370 | 10 | 2.7  (1.3-4.9) | 370 | 0 | - | 2083 | 227 | 10.9  (9.6-12.4) | 4 | 0.2  (0.1-0.5) |
| Rabaul | 0 | 0 | - | 1 | 0 | - | 100 | 0 | - | 0 |  |
| **Total** | **694** | **20** | **2.8**  **(1.8-4.4)** | **695** | **0** | **-** | **3917** | **352** | **9.0 (8.1-9.9)** | **14** | **0.4 (0.2-0.6)** |

**Table B. Total CFA and MF prevalence in 6- 9 year age-group post-MDA for each village.**

| **District** | **Village** | **N** | **F (N)** | **F (%)** | **CFA (N)** | **CFA % (95% CI)** | **MF (N)** | **MF % (95% CI)** |
| --- | --- | --- | --- | --- | --- | --- | --- | --- |
| Kokopo | Ganai | 0 |  |  |  |  |  |  |
|  | Kababia | 0 |  |  |  |  |  |  |
|  | Kabatira | 47 | 22 | 46.81 | 0 | - | 0 |  |
|  | Karawara | 47 | 19 | 40.43 | 5 | 10.64 (3.6-23.1) | 0 |  |
|  | Mualim | 48 | 29 | 60.42 | 0 |  |  |  |
|  | Palpal | 0 |  |  |  |  |  |  |
|  | Ralauna | 0 |  |  |  |  |  |  |
|  | Utuwan | 45 | 26 | 57.78 | 4 | 8.89 (2.5-21.2) | 0 |  |
|  | Virian | 44 | 21 | 47.73 | 0 |  | 0 |  |
| Gazelle | Kamanaka | 0 |  |  |  |  |  |  |
|  | Karo | 46 | 21 | 45.65 | 0 |  | 0 |  |
|  | Lan | 47 | 28 | 59.57 | 1 | 2.13 (0.1-11.3) | 0 |  |
|  | Matanaku | 0 |  |  |  |  |  |  |
|  | Mobilim | 0 |  |  |  |  |  |  |
|  | Napapar1 | 0 |  |  |  |  |  |  |
|  | Puktas | 0 |  |  |  |  |  |  |
|  | Ragaga | 0 |  |  |  |  |  |  |
|  | Ulak | 0 |  |  |  |  |  |  |
|  | Vunapala | 0 |  |  |  |  |  |  |
|  | Warakind | 0 |  |  |  |  |  |  |
|  | Wuatam | 0 |  |  |  |  |  |  |
| Pomio | Awatka | 0 |  |  |  |  |  |  |
|  | Bogotata | 21 | 8 | 38.10 | 1 | 4.76 (0.1-23.8) | 0 |  |
|  | Buka | 17 | 15 | 88.24 | 1 | 5.88 (0.2-28.7) | 0 |  |
|  | Bulus | 0 |  |  |  |  |  |  |
|  | Gar | 40 | 22 | 55.00 | 0 |  | 0 |  |
|  | Gumgum | 0 |  |  |  |  |  |  |
|  | Hoiya | 41 | 19 | 46.34 | 2 | 4.88 (0.6-16.5) | 0 |  |
|  | Illi | 0 |  |  |  |  |  |  |
|  | Ivai | 0 |  |  |  |  |  |  |
|  | Karlai | 0 |  |  |  |  |  |  |
|  | Kaukum | 49 | 20 | 40.82 | 3 | 6.12 (1.3-16.9) | 0 |  |
|  | Kavudemk | 0 |  |  |  |  |  |  |
|  | Kolai | 43 | 17 | 39.53 | 0 | - | 0 |  |
|  | Lamarian | 0 |  |  |  |  |  |  |
|  | Lat | 53 | 16 | 30.19 | 0 | - | 0 |  |
|  | Long | 0 |  |  |  |  |  |  |
|  | Masarau | 0 |  |  |  |  |  |  |
|  | Milim | 11 | 6 | 54.55 | 0 | - | 0 |  |
|  | Muu | 0 |  |  |  |  |  |  |
|  | PomComSc | 0 |  |  |  |  |  |  |
|  | Pulpul | 1 | 1 | 100.00 | 0 | - | 0 |  |
|  | Rainut | 18 | 8 | 44.44 | 0 | - | 0 |  |
|  | Riete | 0 |  |  |  |  |  |  |
|  | Sivauna | 47 | 28 | 59.57 | 3 | 6.52 (1.4-17.9) | 0 |  |
|  | Tokai | 29 | 13 | 44.83 | 0 |  | 0 |  |

**Table C. Total CFA and MF prevalence in >18 year age-group post-MDA for each village.**

| **District** | **Village** | **N** | **Female (N)** | **Female (%)** | **CFA (N)** | **CFA % (95% CI)** | **Mf (N)** | **Mf % (95% CI)** |
| --- | --- | --- | --- | --- | --- | --- | --- | --- |
| Kokopo | Ganai | 100 | 69 | 69.00 | 16 | 16.16 (9.5-24.9) |  |  |
|  | Kababia | 100 | 57 | 57.00 | 6 | 6 (2.2-12.6) |  |  |
|  | Kabatira | 53 | 38 | 71.70 | 6 | 11.32 (4.3-23.0) | 1 | 1.89 (0.1-10.1) |
|  | Karawara | 53 | 28 | 52.83 | 17 | 32.69 (20.3-47.1) | 2 | 3.77 (0.5-12.9) |
|  | Mualim | 51 | 30 | 58.82 | 4 | 7.84 (2.2-18.9) |  |  |
|  | Palpal | 101 | 75 | 74.26 | 7 | 6.93 (2.8-13.8) |  |  |
|  | Ralauna | 58 | 37 | 63.79 | 0 |  |  |  |
|  | Utuwan | 55 | 31 | 56.36 | 32 | 58.18 (44.1-71.4) | 7 | 12.73 (5.3-24.5) |
|  | Virian | 56 | 42 | 75.00 | 3 | 5.36 (1.1-14.9) |  |  |
| Gazelle | Kamanaka | 100 | 57 | 57.00 | 0 |  |  |  |
|  | Karo | 54 | 31 | 57.41 | 1 | 1.85 (0.1-9.9) |  |  |
|  | Lan | 107 | 58 | 54.21 | 20 | 18.69 (11.8-27.4) | 1 | 0.93 (0.0-5.1) |
|  | Matanaku | 102 | 56 | 54.90 | 3 | 2.94 (0.6-8.4) |  |  |
|  | Mobilim | 100 | 35 | 35.00 | 4 | 4 (1.1-9.9) |  |  |
|  | Napapar1 | 98 | 55 | 56.12 | 2 | 2.04 (0.3-7.2) |  |  |
|  | Puktas | 100 | 55 | 55.00 | 4 | 4 (1.1-9.9) |  |  |
|  | Ragaga | 100 | 61 | 61.00 | 6 | 6.06 (2.3-12.7) |  |  |
|  | Ulak | 99 | 52 | 52.53 | 3 | 3.03 (0.6-8.6) |  |  |
|  | Vunapala | 102 | 63 | 61.76 | 1 | 0.98 (0.0-5.3) |  |  |
|  | Warakind | 100 | 52 | 52.00 | 7 | 7 (2.9-13.9) |  |  |
|  | Wuatam | 99 | 25 | 25.25 | 2 | 2.04 (0.3-7.2) |  |  |
| Pomio | Awatka | 100 | 55 | 55.00 | 4 | 4 (1.1-9.9) |  |  |
|  | Bogotata | 82 | 54 | 65.85 | 12 | 14.81 (7.9-24.5) |  |  |
|  | Buka | 81 | 47 | 58.02 | 23 | 28.4 (18.9-39.5) |  |  |
|  | Bulus | 98 | 55 | 56.12 | 19 | 19.39 (12.1-28.6) |  |  |
|  | Gar | 59 | 40 | 67.80 | 0 |  |  |  |
|  | Gumgum | 100 | 45 | 45.00 | 6 | 6 (2.2-12.6) | 1 | 1.0 (0.0-5.5) |
|  | Hoiya | 58 | 36 | 62.07 | 15 | 25.86 (15.3-39.0) |  |  |
|  | Illi | 100 | 48 | 48.00 | 0 |  |  |  |
|  | Ivai | 98 | 74 | 75.51 | 6 | 6.12 (2.3-12.9) | 1 | 1.02 (0.0-5.6) |
|  | Karlai | 100 | 46 | 46.00 | 17 | 17 (10.2-25.8) |  |  |
|  | Kaukum | 51 | 25 | 49.02 | 13 | 25.49 (14.3-39.6) |  |  |
|  | Kavudemk | 100 | 56 | 56.00 | 12 | 12.12 (6.4-20.2) |  |  |
|  | Kolai | 50 | 34 | 68.00 | 4 | 8 (2.2-19.2) |  |  |
|  | Lamarian | 99 | 53 | 53.54 | 11 | 11.11 (5.7-19.0) |  |  |
|  | Lat | 107 | 58 | 54.21 | 20 | 18.69 (11.8-27.4) | 1 | 0.93 (0.0-5.1) |
|  | Long | 99 | 36 | 36.36 | 14 | 14.29 (8.0-22.8) | 1 | 1.02 (0.0-5.6) |
|  | Masarau | 77 | 50 | 64.94 | 5 | 6.49 (2.1-14.5) |  |  |
|  | Milim | 88 | 53 | 60.23 | 6 | 6.82 (2.5-14.3) |  |  |
|  | Muu | 99 | 48 | 48.48 | 8 | 8.08 (3.6-15.3) |  |  |
|  | PomComSc | 82 | 47 | 57.32 | 0 |  |  |  |
|  | Pulpul | 86 | 54 | 62.79 | 6 | 7.06 (2.6-14.7) |  |  |
|  | Rainut | 50 | 24 | 48.00 | 2 | 4 (0.5-13.7) |  |  |
|  | Riete | 100 | 51 | 51.00 | 2 | 2 (0.2-7.0) |  |  |
|  | Sivauna | 49 | 28 | 57.14 | 6 | 12.24 (4.6-24.8) |  |  |
|  | Tokai | 70 | 45 | 64.29 | 16 | 22.86 (13.7-34.4) |  |  |
| Rabaul | Tavui | 100 | 57 | 57.58 | 0 | - |  |  |
