# Supplementary material for: Alternative approaches for monitoring and evaluation of lymphatic filariasis following mass drug treatment with ivermectin, diethylcarbamazine and albendazole in East New Britain Province, Papua New Guinea
Source: PLoS Negl Trop Dis. 2025 Jan 27;19(1):e0012128. doi: 10.1371/journal.pntd.0012128 (PMC11798438; doi:10.1371/journal.pntd.0012128)
Supplement: S1 Fig — The numbers above the bars represent the sample size for each stratum and sex. The dashed line indicates the 80% recommended coverage goal for a highly efficient MDA. (DOCX) [file pntd.0012128.s008.docx]

**S1 Fig**. Results of a coverage survey of 45 clusters and 450 households representing 2,598 children and adults. The numbers above the bars represent the sample size for each stratum and sex. The dashed line indicates the 80% recommended coverage goal for a highly efficient MDA.
